# Phenotypic adaptation and genomic variation of *Kandelia obovata* associated with its northern introduction along southeastern coast of China

**DOI:** 10.3389/fpls.2025.1512620

**Published:** 2025-03-26

**Authors:** Jiaqi Zhang, Sheng Ouyang, Xingxing Cai, Sheng Yang, Qiuxia Chen, Ji Yang, Zhiping Song, Wenju Zhang, Yuguo Wang, Yan Zhu, Peng Nan

**Affiliations:** ^1^ School of Life Sciences, Fudan University, Shanghai, China; ^2^ State Key Laboratory of Wetland Conservation and Restoration, National Observations and Research Station for Wetland Ecosystems of the Yangtze Estuary, Ministry of Education Key Laboratory for Biodiversity Science and Ecological Engineering, and Institute of Eco-Chongming, Shanghai, China; ^3^ Wenzhou Key Laboratory of Resource Plant Innovation and Utilization, Zhejiang Institute of Subtropical Crops, Zhejiang Academy of Agricultural Sciences, Wenzhou, Zhejiang, China

**Keywords:** *Kandelia obovata*, phenotypic adaption, genomic variation, northern introduction, common garden experiment

## Abstract

**Introduction:**

Mangroves play a crucial role within coastal wetland ecosystems, with *Kandelia obovata* frequently utilized for introduction studies and cultivation research. Investigating the rapid adaptability of *K. obovata* across diverse environmental conditions offers valuable insights into how mangroves can effectively acclimate to global climate fluctuations.

**Methods:**

In this study, following a common gardenexperiment, we investigated variations in morphological traits between twodistinct populations of *K. obovata*, Quanzhou (QZ) and Wenzhou (WZ),originating from the same introduction site Zhangzhou (ZZ). Then we performed the whole-genome resequencing on multiple populations along the southern coast of China to assess genetic divergence and diversity patterns in response to environmental factors.

**Results:**

Our findings have uncovered divergent growth-defense trade-off mechanisms employed by these two populations when exposed to varying minimal temperatures in the coldest month within their respective habitats. Moreover, our observations have revealed discernible genetic divergence during the process of environmental acclimatization. Subsequent whole-genome re-sequencing have unveiled a significant decrease in genetic diversity within the northernmost population, suggesting that temperature plays a primary role in shaping genetic variability within the *K. obovata* species.

**Discussion:**

These findings present new evidence for the rapid adaptation of *K. obovata* and contributes to our understanding of environmental adaptation characteristics during its introduction to northern regions, which holds significant implications for the conservation and sustainable development of mangroves.

## 1 Introduction

Given the escalating severity of global climate change, understanding the mechanisms by which organisms adapt to environmental fluctuations has emerged as a pivotal area of scientific investigation in evolutionary biology (Bjorkman et al., 2017). The adaptability of plants stands as a critical determinant influencing their survival capacity within an ever-changing climatic milieu (Foyer and Kranner, 2023). Exploring the adaptive responses exhibited by plants towards diverse environmental alterations can provide valuable insights into their potential coping strategies amidst global climate change. Mangroves represent arboreal ecosystems thriving in tropical and subtropical intertidal zones, characterized by remarkable biodiversity and recognized as one of the foremost carbon-sequestering habitats worldwide. They play indispensable roles in storm resistance, coastal protection, and preservation of coastal ecological equilibrium (Li et al., 2024). Temperature serves as the primary environmental factor delimiting the geographical distribution of mangroves, making them among the most vulnerable groups affected by global climate change (Chen et al., 2017). Consequently, mangrove plants offer ideal experimental subjects for investigating rapid plant responses to global climate change. However, due to intensified human activities and global climate change, mangrove forests are experiencing considerable decline worldwide. Consequently, artificial plantation of mangroves has emerged as an essential strategy for ecosystem restoration (Ellison et al., 2020). There is abundant evidence suggesting that introduced mangrove forests in new environments often undergo adaptive evolution to cope with selection pressures specific to these novel habitats (Miryeganeh, 2022).

Plants adopt phenotypic plasticity and genetic differentiation as two distinct strategies to adapt to their environment (Boquete et al., 2021; Bastias et al., 2024). During the course of evolution, plants must balance these two approaches. Initially, a plant species may utilize plasticity to mitigate natural selection pressure when colonizing a new environment. However, with time and expansion of its geographic range, plant populations may undergo genetic differentiation. In this process, plants can develop various ecotypic traits to adapt to diverse climatic and geographical conditions (Baythavong and Stanton, 2010). Studies have demonstrated that successful invasion by *Brachypodium sylvatium* is driven by genetic variation rather than phenotypic plasticity induced by the environment (Marchini et al., 2019). A common garden experiment (CGE) can eliminate the influence of environmental factors from provenances on results and assess whether plant populations from different locations have genetic differentiation (Zhao et al., 2021).

Research has revealed that *K. obovata*, a member of the Rhizophoraceae family, is a widely distributed mangrove species in China renowned for its ability to thrive in high latitudes and withstand cold temperatures (Su et al., 2019). Previous study has indicated the presence of significant genetic differentiation and variation within *K. obovata* populations along the southeastern coast of China (Zhao et al., 2024). These populations exhibit substantial genetic diversity and may serve as key centers of diversity for Asian mangroves. The genetic diversity of mangroves has been extensively investigated using molecular marker techniques such as Sequence-Related Amplified Polymorphism (SRAP) and Simple Sequence Repeats (SSR) in previous studies (Lu et al., 2021). However, with the rapid advancement of sequencing technology, a chromosome-level reference genome for *K. obovata* was published in 2020 (Hu et al., 2020). By employing resequencing and bioinformatics methods, studying the genomic level differences and adaptive evolution between populations enables us to comprehend the genetic mechanisms underlying organisms’ adaptation to selection and identify crucial candidate genes. This provides a theoretical foundation for the rational utilization of germplasm resources (Lu et al., 2023).

As part of the exploration work on mangrove protection and introduction in China, *K. obovata* was introduced from the Jiulong River Estuary Mangrove Provincial Nature Reserve in Zhangzhou city (**ZZ**), Fujian Province (117°92’E, 24°46’N) to the Luoyang River Mangrove Nature Reserve in Quanzhou Bay, Quanzhou city (**QZ**), Fujian Province (118°59’E, 24°59’N) in 2003. Subsequently, it was further introduced to Longgang Aojiang Estuary Mangrove, Wenzhou city (**WZ**), Zhejiang Province (120°96’E, 28°12’N) in 2005. After approximately two decades since its original habitat to the introduction sites, significant phenotypic variations have been observed in these two populations of *K. obovata*. This study aims to investigate whether these differences are associated with genetic background through CGE. Additionally, using whole-genome re-sequencing (WGRS) technology, this study analyzes and compares the levels of genetic diversity between populations of *K. obovata* from its original habitat and introduction sites to unravel their phylogenetic relationships and genetic structure. These findings will provide genetic data support for identifying and utilizing germplasm resources of *K. obovata*. Furthermore, this study explores candidate genes under strong selective pressure across different geographical environments and predicts cis-regulatory elements within their promoter regions to elucidate the impact of environmental adaptation on population differentiation of *K. obovata*. This provides a theoretical basis for understanding rapid ecological adaptation mechanisms of *K. obovata* towards environmental changes and strengthens ecological research on global climate response and adaptability of mangroves. It is also significant for mangrove conservation, new variety breeding, and serves as a reference for studying mechanisms by which plants rapidly adapt to environmental changes.

## 2 Materials and methods

### 2.1 Common garden experiment

In May 2019, we acquired approximately 20,000 hypocotyls of *K. obovata* from two distinct locations: the Luoyang River Mangrove Nature Reserve in Quanzhou Bay, Quanzhou City (QZ), Fujian Province, and the Longgang Aojiang Estuary Mangrove in Wenzhou City (WZ), Zhejiang Province. These individuals were then transplanted into plastic pots at the Mangrove Base of Nanhui Dongtan, Shanghai (SH) (121°97’E,30°90’N) for cultivation under CGE conditions. The objective was to assess the overwintering survival rate of *K. obovata* seedlings under natural conditions (**Supplementary Figure 1**). In May 2021, we additionally acquired mature hypocotyls of *K. obovata* from both QZ and WZ. We conducted a random sampling of 30 healthy hypocotyls from each population and recorded their hypocotyl length (HL), hypocotyl weight (HW), and hypocotyl diameter (HD). Subsequently, we carefully selected 1,000 individuals with comparable weights, lengths, and sizes from each population. These individuals were also transplanted into plastic pots in SH for CGE cultivation, with the purpose of subsequent phenotypic observations. The Nanhui Dongtan, located in the Yangtze estuary, is the largest and most extensive coastal wetland in the region. It experiences a subtropical monsoon climate characterized by moderate temperatures and high humidity levels. This area exhibits features of both monsoon and maritime climates. With an average annual temperature ranging from 15 to 16 °C, it has recorded its highest temperature at 37.3 °C and lowest temperature at -7.9 °C.

### 2.2 Determination of morphological traits

After 18 months of growth in CGE, a total of 30 robust and healthy plants were randomly selected from both the QZ and WZ populations for comprehensive measurement of plant morphological traits. The assessment covered the following parameters: plant height (PH), basal diameter (BD), crown width (W), leaf number (LN), and branch number (BN). Thirty intact leaves were carefully collected from the upper branches of both QZ and WZ populations, thoroughly rinsed with tap water, and then precisely weighed to determine their fresh weight (LFW). After being soaked in distilled water for 12 hours followed by thorough drying to eliminate surface moisture, the leaves were reweighed to obtain their saturated fresh weight (LSFW). Subsequently, scanned images of the collected leaves were analyzed using ImageJ software (1.53t) (Schneider et al., 2012) to calculate various parameters such as leaf length (LL), leaf width (LW), leaf perimeter (LP), leaf area (LA), leaf shape index (LSI = LL/LW). The collected samples were placed in trays and subjected to constant drying at 60°C for 48 hours until reaching a stable weight. The dry weight (DW) was measured to compute relative water content (RWC=(LFW-DW)/(LSFW-DW)) and leaf dry matter content (LDMC=DW/LSFW). Specific leaf area (SLA) is calculated as LA/DW.

### 2.3 Leaf anatomical structure

We employed the Safranin-O/Fast green staining method to prepare *K. obovata* samples. Paraffin sections were made with the second leaf from the top down, and ImageJ software (1.53t) was utilized for quantifying the thickness of various leaf components: leaf thickness (Lt), upper cuticle layer thickness (UCu), upper epidermis thickness (UEp), upper hypodermis thickness (UHy), upper palisade tissue thickness (UPt), spongy tissue thickness (St), lower cuticle layer thickness (LCu), lower epidermis thickness (LEp), lower hypodermis thickness (LHy) as well as lower palisade tissue thickness (LPt). The ratio of palisade to spongy tissues (P/S=Pt/St) was calculated along with cell tense ratio (CTR=Pt/LT) and spongy ratio (SR=St/LT).

### 2.4 Assessment of stomatal characteristics

30 leaf samples were selected from both the QZ and WZ populations in CGE. Temporary slides of the lower epidermis stomata of *K. obovata* leaves were prepared using the nail polish imprinting method (Pathoumthong et al., 2023), observed, and photographed under a 10x optical microscope. ImageJ software (1.53t) was utilized to quantify the stomata number (SN), stomatal area (SA), and stomatal density (SD) per view field. 10-15 view fields were examined in each sample, and the results were averaged.

### 2.5 Determination of cold resistance

We measured the daily minimum temperature changes at Nanhui Dongtan Mangrove Base in Shanghai during the coldest months of 2020 and 2021 as shown in **Supplementary Figure 2**. Despite a warm winter in Shanghai in 2020, an extended period of extreme low temperatures (-7°C) occurred from December 29, 2020 to January 2, 2021. The overwinter survival rates of mangrove plants in their natural environment at the base were separately recorded on January 21, 2020 (without extreme low temperatures) and January 5, 2021 (during the cold wave with extreme low temperatures). The data was analyzed using Microsoft Excel (2021) software. Non-paired Student’s *t*-test was employed to compare significant differences among different populations in various indicator values. Additionally, a Chi-square test with Yates’ continuity correction was used to assess the differences in overwinter survival rates between the WZ and QZ populations under different temperature conditions. A significance level of *p*>0.05 indicated non-significance; a range of 0.01<*p*<0.05 denoted significance; while *p*<0.01 represented high significance levels. R (4.2.2), Rstudio (2023.03.0 + 386) ggplot2 package and GraphPad Prism (8) software were utilized for graphical representation purposes.

### 2.6 Whole-genome re-sequencing

In mid to late July 2023, mature *K. obovata* leaves samples were collected from four locations: Dongzhaigang Mangrove Nature Reserve (DZG) in Haikou City, Hainan Province (110°58’E,19°95’N); Jiulong River Estuary Mangrove Provincial Nature Reserve in Zhangzhou City (ZZ), Fujian Province (117°92’E,24°46 N); Luoyang River Mangrove Nature Reserve in Quanzhou Bay, Quanzhou City (QZ), Fujian Province (118°59’E,24°91’N); and Longgang Aojiang Estuary Mangrove in Wenzhou City (WZ), Zhejiang Province (120°96’E,28°12’N) (**Supplementary Figure 1**). A total of 40 samples were collected, comprising 10 mature *K. obovata* leaves from each location, with a minimum distance of over 50 meters maintained between the collection points.

The CTAB method was employed to extract DNA from the leaf samples. Only high-quality DNA samples (OD_260_/_280_ = 1.8~2.0, OD_260_/_230_ = 2.0) were utilized for constructing the sequencing library. A total of 0.5 μg of DNA per sample served as input material for the preparation of the DNA library. The Truseq Nano DNA HT Sample Prep Kit (Illumina USA) was used to generate the sequencing library in accordance with the manufacturer’s recommendations, and index codes were assigned to each sample. In brief, genomic DNA samples were sonicated to obtain fragments with a size of 350 bp, followed by end-polishing, A-tailing, and ligation with full-length adapters suitable for Illumina sequencing technology; this was succeeded by additional PCR amplification steps. After purification of PCR products using the AMPure XP system, libraries underwent size distribution analysis via Agilent 2100 Bioanalyzer and quantification through real-time PCR (3nM). Finally, paired-end DNA-seq sequencing libraries were sequenced on an Illumina NovaSeq system at Shanghai Majorbio Bio-pharm Technology Co., Ltd.

### 2.7 Variant discovery

The raw reads of low quality (mean phred score < 20), which included reads containing adapter contamination and unrecognizable nucleotides (N base > 10), were trimmed or discarded using Fastp software (0.23.2) (Chen et al., 2018). After trimming, the reads were mapped to the reference genome (https://bigd.big.ac.cn/gwh/Assembly/990/show) using BWAMEME software (1.0.5) (Jung and Han, 2022) with default mapping parameters. The alignment bam files were sorted by SAMtools (1.15.1) (Li et al., 2009) and PCR duplicates were marked using MarkDuplicated as part of the modified GATK Best Practice pipeline (4.3.0.0) (Mckenna et al., 2010). Base quality recalibration was performed, followed by germline variant calling for Single Nucleotide Polymorphisms (SNPs) across all samples using Haplotyper and Gvcftyper programs in Sentieon genomics tools (202112.07) (Freed et al., 2017). Variants were filtered according to standard hard filtering parameters based on GATK Best Practices pipeline (4.3.0.0), and annotated using SnpEff (5.1d) (Cingolani et al., 2012). Subsequently, several filtering steps were applied to reduce false positives for SNPs and genotype calling using VCFtools software (0.1.16) (Danecek et al., 2011): (if) SNPs with more than two alleles were removed, (ii) SNPs with mean depth values less than 4 across all samples were removed, (iii) SNPs with minor allele frequency < 0.05 were removed, (iv) Only SNPs that could be genotyped in at least 70% of the samples were retained, (v) For population structure analysis, SNPs showing linkage disequilibrium patterns were pruned using Plink software (1.90b6.20) (Purcell et al., 2007).

### 2.8 Genetic diversity analysis

Based on filtered vs. files, we calculated observed heterozygosity (Ho), expected heterozygosity (He) and nucleotide diversity (π) using the populations module in the software Stacks (2.64) (Catchen et al., 2013). Additionally, GenAlEx (6.5) (Peakall and Smouse, 2012) was employed to determine genetic diversity parameters such as Polymorphic Information Content (PIC) and Shannon’s Information Index. These parameters were utilized to evaluate the level of genetic diversity in four populations of *K. obovata* studied here. A higher value for these genetic diversity parameters indicates a greater level of genetic diversity within the population.

The Genetic Differentiation Index (*F*_ST_) between populations was calculated using the populations module in the Stacks software (2.64) (Catchen et al., 2013). *F*_ST_ values were obtained through pairwise comparisons among all populations to evaluate the extent of genetic differentiation between two populations. The level of genetic differentiation can be determined based on a range of *F*_ST_ values: 0~0.05 indicates an extremely low degree of genetic differentiation between populations; 0.05~0.15 suggests a moderate level of genetic differentiation; 0.15~0.25 signifies a considerable degree of genetic differentiation between populations; and when *F*_ST_ > 0.25, it indicates a high level of genetic differentiation among populations (Wright, 1978).

### 2.9 Phylogenetic analyses

The Maximum Likelihood phylogenetic tree was constructed using IQ-TREE2 software (2.1.2) (Minh et al., 2020). The ML analyses were performed on the pruned SNP sites employing IQ-TREE2 with GTR+I+G4 model and 1000 bootstraps.

The unsupervised maximum-likelihood clustering algorithm implemented in ADMIXTURE (1.3.0) (Alexander et al., 2009) was used to cluster each genome in the investigated populations of *K. obovata*. Initial clustering was performed for K = 1 to K = 20 ancestral clusters using default settings. The optimal value of K was determined based on the cross-validation error (CV error), and the genetic structure corresponding to this optimal value of K was outputted as the final result. To enhance the accuracy of initial clustering, pruned SNPs were utilized in the structural analysis.

To visualize the genetic relationships among samples, we conducted principal component analysis (PCA) based on pruned SNPs using Plink (1.90b6.20) (Purcell et al., 2007).

### 2.10 Screening for selective sweep and identification of positively selected genes

Following the sliding window strategy, we utilized PIXY software (1.2.7.beta1) (Korunes and Samuk, 2021) to perform segmentation and calculation of *F*_ST_ as well as π between QZ and WZ populations. The window size was set at 10 Mb with a step size of 10 kb. To strengthen the analysis of positive selection, we employed a permutation test to account for random differentiation that may occur between populations in the absence of selection pressure. Specifically, we performed 1,000 permutation tests by randomly shuffling the population labels and recalculating the *F*_ST_ distribution for each 10 kb window. This approach allowed us to simulate different population structures under the null hypothesis of no selection. The 95^th^ percentile of the permutation distribution was used as a baseline to determine whether the observed *F*_ST_ values were significantly higher than expected by chance. Finally, we employed a screening criterion where regions with *F*_ST_ values in the top 5% were identified as potential candidate regions distinguishing QZ from WZ populations. Furthermore, our selective sweep analysis combined both *F*_ST_ and π values. Specifically, regions of the genome exceeding the top 5% threshold, along with regions exhibiting extremely high π ratios (π_QZ_/π_WZ_), were considered as potential areas displaying strong signals of selection scanning for WZ population. Sequence Toolkit in TBtools (2.112) (Chen et al., 2020) was used to associate the selected region with candidate genes to obtain the positively selected genes of WZ population.

The TBtools software (2.112) was also employed to conduct Gene Ontology (GO) and Kyoto Encyclopedia of Genes and Genomes (KEGG) enrichment analysis on the identified regions from the QZ and WZ populations. Significance of enrichment was determined when *p*-value < 0.05 for both GO and KEGG pathways. Visualization was performed using the qqman and ggplot2 packages in R (4.2.2) and Rstudio (2023.03.0 + 386) software.

### 2.11 Analysis of cis-element in promoters

After extracting the 2,000 bp upstream sequence of the positive selection gene in the WZ population using TBtools software (2.112), we conducted promoter cis-element analysis through the PlantCare database (Lescot et al., 2002).

### 2.12 Integrated analysis of transcriptome and genome data

We retrieved previously generated transcriptome data (Zhang et al., 2024), and intersected the differentially expressed genes (DEGs) under cold stress from both populations with 40 candidate positively selected genes identified from the genome based on *F*_ST_ and π values. These genes were further analyzed for transcriptome expression profiles and promoter elements. Heatmap visualization was performed using ggplot2 and pheatmap packages in R (4.2.2) and RStudio (2023.03.0 + 386). Transcriptional levels and cis-elements were visualized using TBtools Simple BioSequence Viewer (2.112) and GraphPad Prism (8).

## 3 Results

### 3.1 Morphological differences between two populations of *K. obovata*

**Figure 1:**
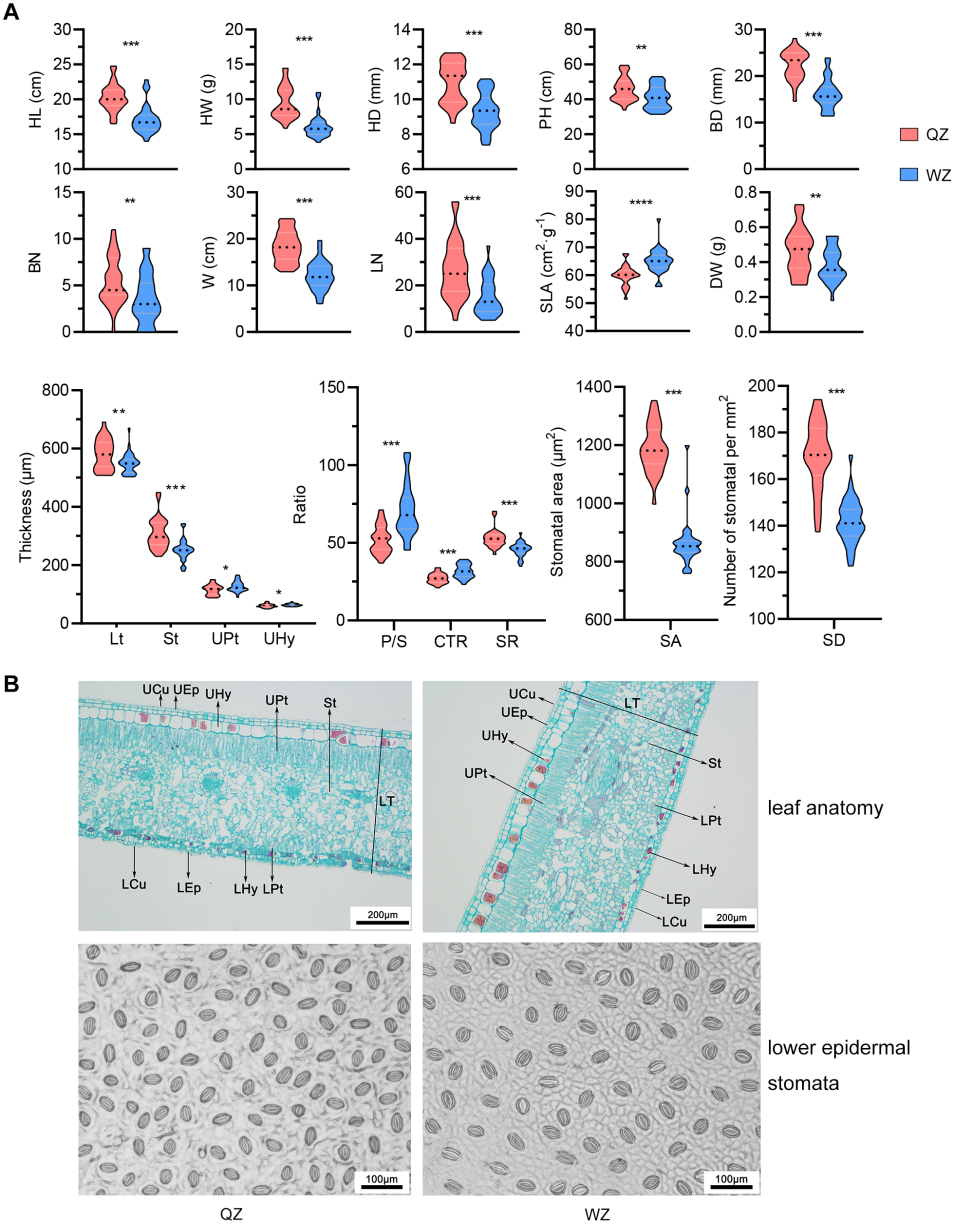
Differences in morphology and leaf traits between two populations of K. *obovata*. **(A)** Comparison of morphological traits between two populations of *K*. *obovata*. HL, hypocotyl length; HW, hypocotyl weight; HD, hypocotyl diameter; PH, plant height; BD, basal diameter; BN, branch number; W, crown width; LN, leaf number; SLA, specific leaf area; DW, dry weight; Lt, leaf thickness; St, spongy tissue thickness; UPt, upper palisade tissue; UHy, upper hypodermis; P/S, palisade-spongy tissue ratio; CTR, cell tense ratio; SR, spongy ratio; SA, stomatal area; SD, stomatal density. * represents *p*<0.05; ** represents *p*<0.01, *** *p* represents <0.001. **(B)** Comparison of leaf anatomy and lower epidermal stomata between two populations of *K*. *obovata*. UCu and LCu, upper and lower cuticles; UEp and LEp, upper and lower epidermis; UHy and LHy, upper and lower hypodermis; UPt and LPt, upper and lower palisade tissue; St, spongy tissue; LT, leaf thickness.

In comparison to the QZ population, the WZ population’s hypocotyls exhibit relatively lower values for physiological parameters (**Supplementary Table 1**). There are statistically significant differences (*p*<0.05, non-paired Student’s *t*-test) in HL, HW, HD, PH, BD, LN, and BN, as well as W between these two populations. Moreover, significant differences between the two populations (*p*<0.05, non-paired Student’s *t*-test) are also observed in SLA, and DW (**Figure 1**). However, no notable differences are found in LW, LSI, LA, RWC or LDMC.

### 3.2 Disparities in leaf anatomical structure and stomatal characteristics between two *K. obovata* Populations

The cross-sectional images of *K. obovata* reveal a leaf structure comprising upper and lower cuticles, epidermis, hypodermis, palisade tissue, and spongy tissue. Both the upper and lower epidermis consist of a single layer of cells, while the hypodermis consists of two cell layers—the first layer being smaller without tannins and the second layer larger containing tannins. The densely arranged palisade tissue has a thicker upper layer than the lower layer. The spongy tissue, which accommodates vascular bundles, displays a looser arrangement (**Figures 1A, B**). Furthermore, the Lt, St, and SR are significantly greater in the QZ population compared to those in the WZ population (*p*<0.05, non-paired Student’s *t*-test). Conversely, the WZ population exhibits significantly higher thickness in UHy, UPt, P/S, and CTR compared to the QZ population (*p*<0.05, non-paired Student’s *t*-test). Stomata of *K. obovata* are predominantly distributed in the lower epidermis (**Figure 1B**). The SA is significantly smaller in QZ population than that in WZ population (*p*<0.05, non-paired Student’s *t*-test), while SD is significantly larger than that in WZ population (*p*<0.05, non-paired Student’s *t*-test) (**Figure 1A**).

### 3.3 Variation in Overwintering Survival Rate between Two *K. obovata* Populations under Natural Conditions

One of the primary constraints on the northward expansion of *K. obovata* is its susceptibility to cold temperatures. We investigated the overwinter survival rates of both populations under natural conditions following the CGE (**Table 1**). In January 2020 with moderate winter temperatures, the survival rate of *K. obovata* was notably high. Specifically, the natural overwinter survival rate of WZ population was 84.5%, surpassing that of the QZ population (67.3%). Both populations exhibited reduced survival rates under extreme low temperatures in 2021; however, even under such conditions, the WZ population demonstrated a higher winter survival rate (18.6%) compared to that of the QZ population (6.8%). Our findings indicate a significant difference in overwinter survival rates between the two populations both under extreme low temperatures and moderate winter conditions (*p*< 2.2e-16, Chi-square test). The WZ population exhibits superior cold tolerance and better adaptation to the northern CGE environment.

**Table 1:** Natural overwinter survival rates of *K. obovata* in 2020 and 2021.

Populations	numbers	No extreme low temperature (2020)	With extreme low temperature (2021)
WZ	19600	84.5%	18.6%
QZ	19400	67.3%	6.8%
Significant difference	–	*p*<2.2e-16	*p*<2.2e-16

Note that there are significant differences in the survival rates of the WZ and QZ populations in both 2020 and 2021.

### 3.4 WGRS, SNP variation detection and annotation

In this study, we selected samples from four population: DZG as the outgroup, ZZ as the sample from original location, QZ, and WZ. 10 samples were collected from each population, resulting in a total of 40 samples. WGRS was performed on these 40 samples. Following rigorous quality control and raw data filtering, we obtained a total of 92.98 Gb of clean read data with Q30 sequences exceeding 93.36% and GC content ranging from 36.35% to 39.64% (**Supplementary Table 2**). Alignment of the clean data with the reference genome yielded 45.82 Gb of clean data, indicating an average alignment rate of 92.62% between the samples and the reference genome. The genome coverage rate was calculated at 90.14%, with an average sequencing depth of 12.20× and an average coverage rate (≥4×) (**Supplementary Table 3**). The uniform coverage across the entire genome indicated excellent sequencing randomness (**Supplementary Figure 3**).

After filtration, a total of 117,774 high-quality SNPs were identified in the population. Subsequent analysis revealed 69,692 transitions (ts) and 48,082 transversions (tv), resulting in a ts/tv ratio of 1.45 (**Supplementary Table 4**). Statistical analysis based on the annotation information of the *K. obovata genome* indicated that the majority of SNPs were located in intergenic regions, with only approximately 20% found within genic regions (**Supplementary Table 4**). Specifically, whole-genome assessment of WZ and QZ identified a total of 50,330 and 44,418 SNPs respectively, with approximately 20.68% and 19.40% located within genic regions (**Supplementary Figure 4**).

### 3.5 Analysis of genetic diversity and relationship among different populations of *K. obovata*

**Figure 2:**
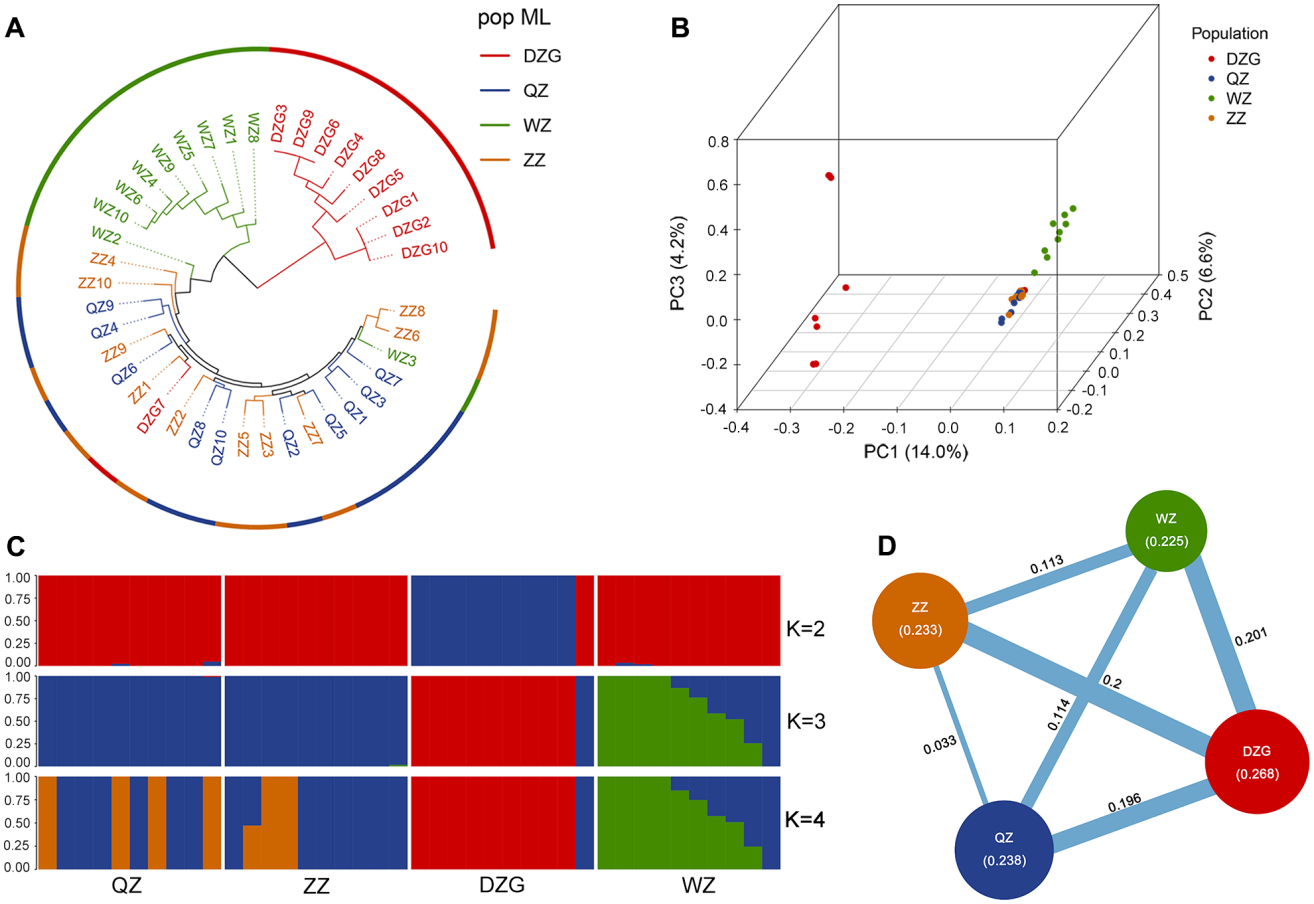
Kinship analysis in different populations of *K*. *obovata*. **(A)** Phylogenetic tree constructed using the maximum likelihood method; **(B)** PCA of the four populations; **(C)** Genetic structure of the four populations based on the WGRS. **(D)** Results of genetic diversity for four populations. The numbers in brackets represent the nucleotide diversity index (π) and the numbers in the lines represent the fixation index (*F*_ST_) between the two populations.

SNP analysis was employed to investigate the genetic relationships among 40 samples. The DZG population was utilized as an outgroup, and a phylogenetic tree was constructed using the maximum likelihood method. The results revealed challenges in distinguishing between the ZZ and QZ populations, indicating low genetic differentiation and close genetic relationship. However, the WZ population exhibited clear differentiation from the ZZ and QZ populations, suggesting substantial genetic divergence between WZ and ZZ/QZ populations (**Figure 2A**). PCA revealed that PC1 effectively distinguished the DZG outgroup from other populations, while PC2 differentiated the WZ population from ZZ and QZ populations. Only a slight difference was observed between ZZ and QZ populations on this principal component, indicating their close genetic relationship (**Figure 2B**). STRUCTURE analysis revealed that a K value of 3 resulted in the lowest error rate of variation coefficient (**Supplementary Figure 5**), leading to the division of the 40 samples into three clusters. This grouping combined QZ and ZZ into one cluster while separating DZG and WZ (**Figure 2C**).

In addition, we computed π, Ho, He, PIC, and Shannon index for each population of *K. obovata* to evaluate their genetic diversity levels. The findings revealed that the DZG population exhibited the highest genetic diversity level among the four *K. obovata* populations, followed by QZ and ZZ; whereas the WZ population displayed the lowest genetic diversity level (**Table 2**). Furthermore, we assessed inter-population genetic differentiation using genetic differentiation index *F*_ST_. The results indicated minimal genetic differentiation between the QZ and ZZ populations, while moderate genetic differentiation was observed between the WZ and QZ as well as ZZ populations (**Figure 2D**).

**Table 2:** Genetic diversity index statistics among *K. obovata* populations.

Pop ID	π	Ho	He	PIC	Shannon Index
DZG	0.268	0.242	0.252	0.199	0.374
QZ	0.238	0.257	0.223	0.177	0.334
ZZ	0.233	0.256	0.219	0.175	0.329
WZ	0.225	0.230	0.211	0.169	0.317

### 3.6 Detection of selection sweep signals and analysis of *cis*-elements in candidate genes

**Figure 3:**
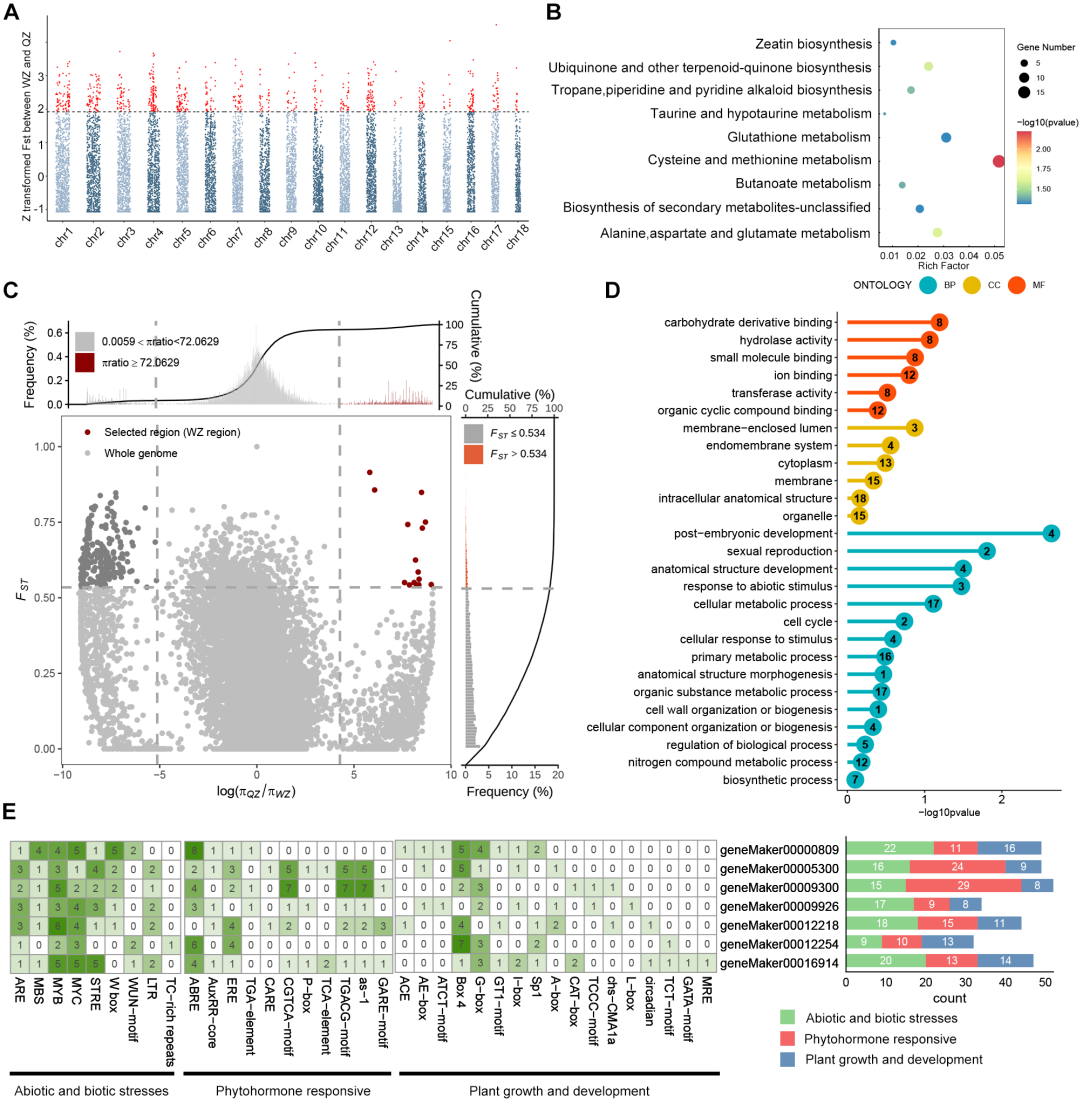
Analysis of selective sweep signals and cis-elements of candidate genes between QZ and WZ populations. **(A)** Manhattan plot of *F*_ST_ distribution with chromosomes. The horizontal axis represents chromosomes, the vertical axis represents *F*_ST_ between QZ and WZ populations, and the dashed line is the threshold line, with the default value of top5%; red loci beyond the threshold line are those with significant selective sweep effects. **(B)** KEGG functional enrichment of genes. The horizontal axis represents rich factors, the vertical axis represents functionally enriched pathways, bubble colors indicate enrichment significance, and bubble sizes indicate the number of genes in the gene set for the candidate gene. **(C)** Selected regions of WZ populations based on the combination of *F*_ST_ and π ratio (QZ/WZ) screened at the top5% level. The horizontal axis represents the ratio of nucleotides, and the vertical axis represents *F*_ST_, with WZ selected regions in red. **(D)** GO functional enrichment analysis under selection signaling of WZ, with the horizontal axis representing significance, the vertical axis representing functionally enriched terms, and the number in the circle denoting the number of selected genes in the gene set. **(E)** Prediction of cis-acting elements in the promoter region of some WZ selected genes. The cis-acting elements were classified into three categories according to their functions, with green indicating abiotic and biotic stresses, red indicating phytohormone responsive, and blue indicating plant growth and development. The numbers in the squares on the left represent the number of cis-elements per gene, and the numbers on the right represent the number of cis-elements with different functions per gene.

Using the 1,000 permutation test, we observed that the 95^th^ percentile of the *F*_ST_ distribution was 0.243 in the absence of selection pressure. In contrast, the real population’s top 5% *F*_ST_ threshold was 0.534 (**Supplementary Figure 6**). The fact that the observed *F*_ST_ value exceeds the 95^th^ percentile of the permutation distribution indicates that these high *F*_ST_ values are not due to random fluctuations and are more likely to reflect genuine selection pressures. Thus, the top 5% regions of *F*_ST_ in *K. obovata* populations from QZ and WZ were identified based on *F*_ST_ scans, resulting in the detection of 785 windows through Manhattan plots. Following alignment with the reference genome annotation file, a total of 1,159 genes were identified (**Figure 3A**, **Supplementary Tables 5**-**7**). KEGG pathway enrichment analysis of these 1,159 candidate genes revealed significant enrichment in 9 pathways (**Supplementary Table 8**). Specifically, 15 genes showed enrichment in cysteine and methionine metabolism, while 9 genes exhibited enrichment in glutathione metabolism, along with 8 genes involved in alanine, aspartate, and glutamate metabolism; additionally, 7 genes were associated with ubiquinone and other terpenoid-quinone biosynthesis (**Figure 3B**).

Furthermore, employing a method that integrates genetic differentiation coefficient (*F*_ST_) and nucleotide diversity (π), we identified 13 regions exhibiting positive selection in WZ, falling within the top 5% of the region between *F*_ST_ and log_2_(π_QZ_/π_WZ_) in WZ (**Figure 3C**). Upon aligning these selected windows with the reference genome annotation file, we determined a total of 40 candidate genes undergoing positive selection in WZ (**Table 3**; **Supplementary Table 9**). GO functional enrichment analysis revealed significant enrichment of biological processes related to stress response, anatomical structure development, sexual reproduction, reproductive development process, and post-embryonic development among the positively selected genes in WZ (**Figure 3D**, **Supplementary Table 10**).

**Table 3:** Candidate genes identified by the top5% *F*_ST_ and π ratio value between WZ and QZ populations (40 genes).

Gene ID	Gene Symbol in Arabidopsis	Description
geneMaker00000121	*LRL12*	Leaf rust 10 disease-resistance locus receptor-like protein kinase-like 1.2
geneMaker00000483	*FH20*	LOW QUALITY PROTEIN: formin-like protein 20 [Populus alba]
geneMaker00000499	*ENO1*	enolase 1, chloroplastic [Manihot esculenta]
geneMaker00000704	*AAE17*	probable acyl-activating enzyme 17, peroxisomal isoform X1 [Populus alba]
geneMaker00000809	*CDPKA*	calcium-dependent protein kinase 10-like [Hevea brasiliensis]
geneMaker00002020	*EPFL2*	EPIDERMAL PATTERNING FACTOR-like protein 2 [Hevea brasiliensis]
geneMaker00002335	*TTL3*	Inactive TPR repeat-containing thioredoxin TTL3
geneMaker00003382	*NOV*	Protein NO VEIN
geneMaker00004151	*EMC1*	ER membrane protein complex subunit 1 [Hevea brasiliensis]
geneMaker00004198	*ELF32*	ELF3-like protein 2
geneMaker00004589	*KTN83*	Katanin p80 WD40 repeat-containing subunit B1 homolog KTN80.3
geneMaker00005300	*PILS6*	protein PIN-LIKES 6-like isoform X2 [Hevea brasiliensis]
geneMaker00005988	*ACA9*	calcium-transporting ATPase 9, plasma membrane-type isoform X1 [Manihot esculenta]
geneMaker00006095	*ILL3*	IAA-amino acid hydrolase ILR1-like 3 [Hevea brasiliensis]
geneMaker00006313	*VOZ1*	Transcription factor VOZ1
geneMaker00006341	*TBL38*	protein trichome birefringence-like 37 [Manihot esculenta]
geneMaker00007113	*PSL5*	probable glucan 1,3-alpha-glucosidase [Hevea brasiliensis]
geneMaker00007422	*DPNP1*	SAL1 phosphatase
geneMaker00007597	*FIGL1*	ATPase family AAA domain-containing protein FIGL1 isoform X1 [Manihot esculenta]
geneMaker00008043	*-*	uncharacterized protein LOC110637836 [Hevea brasiliensis]
geneMaker00008215	*PERK3*	probable receptor-like protein kinase At5g38990 isoform X1 [Hevea brasiliensis]
geneMaker00008285	*-*	uncharacterized protein LOC110637868 isoform X3 [Hevea brasiliensis]
geneMaker00009206	*-*	uncharacterized protein LOC110658574 [Hevea brasiliensis]
geneMaker00009300	*GAUT6*	probable galacturonosyltransferase 6 [Ricinus communis]
geneMaker00009877	*-*	hypothetical protein H0E87_011124 [Populus deltoides]
geneMaker00009926	*-*	PREDICTED: uncharacterized protein LOC105131445 [Populus euphratica]
geneMaker00009969	*TGH*	G patch domain-containing protein TGH [Hevea brasiliensis]
geneMaker00010131	*DHAR2*	Glutathione S-transferase DHAR2
geneMaker00010295	*CPP1*	protein CHAPERONE-LIKE PROTEIN OF POR1, chloroplastic [Jatropha curcas]
geneMaker00010602	*SPO11*	meiotic recombination protein SPO11-1 isoform X3 [Hevea brasiliensis]
geneMaker00011284	*TAZ*	tafazzin [Manihot esculenta]
geneMaker00012218	*SMU1*	suppressor of mec-8 and unc-52 protein homolog 1 [Morus notabilis]
geneMaker00012254	*SYKC*	lysine–tRNA ligase-like isoform X3 [Gossypium australe]
geneMaker00012303	*-*	hypothetical protein MANES_17G067700v8 [Manihot esculenta]
geneMaker00012523	*SUV3M*	DExH-box ATP-dependent RNA helicase DExH16, mitochondrial isoform X2 [Ricinus communis]
geneMaker00015184	*-*	uncharacterized protein LOC18055655 isoform X1 [Citrus clementina]
geneMaker00015878	*AP2A1*	AP-2 complex subunit alpha-1
geneMaker00015905	*LAC4*	laccase-4-like [Hevea brasiliensis]
geneMaker00015962	*DIVARICATA*	transcription factor DIVARICATA [Herrania umbratica]
geneMaker00016914	*SIGF*	RNA polymerase sigma factor sigF, chloroplastic isoform X1 [Manihot esculenta]

We utilized the 2,000 bp upstream sequence as the promoter region for investigating 40 genes exhibiting positive selection in the WZ population. Our analysis revealed an abundance of *cis*-elements associated with non-biological stress responses (**Supplementary Figure 7**). The findings demonstrate widespread presence of stress-responsive elements (STR), low temperature-responsive elements (LTR), MYB-binding sites (MBS) induced by drought, and wound-responsive elements (WUN-motif) within the promoters of positively selected genes in the WZ population. Furthermore, we have observed a wide distribution of diverse cis-elements, including abscisic acid responsive element (ABRE), salicylic acid responsive element (as-1), ethylene responsive element (ERE), jasmonic acid responsive elements (CGTCA-motif and TGACG-motif), as well as numerous light-responsive elements such as Box4, across these promoters. (**Figure 3E**).

### 3.7 Analysis of the relationship between selected genes and cold stress response in the WZ population

**Figure 4:**
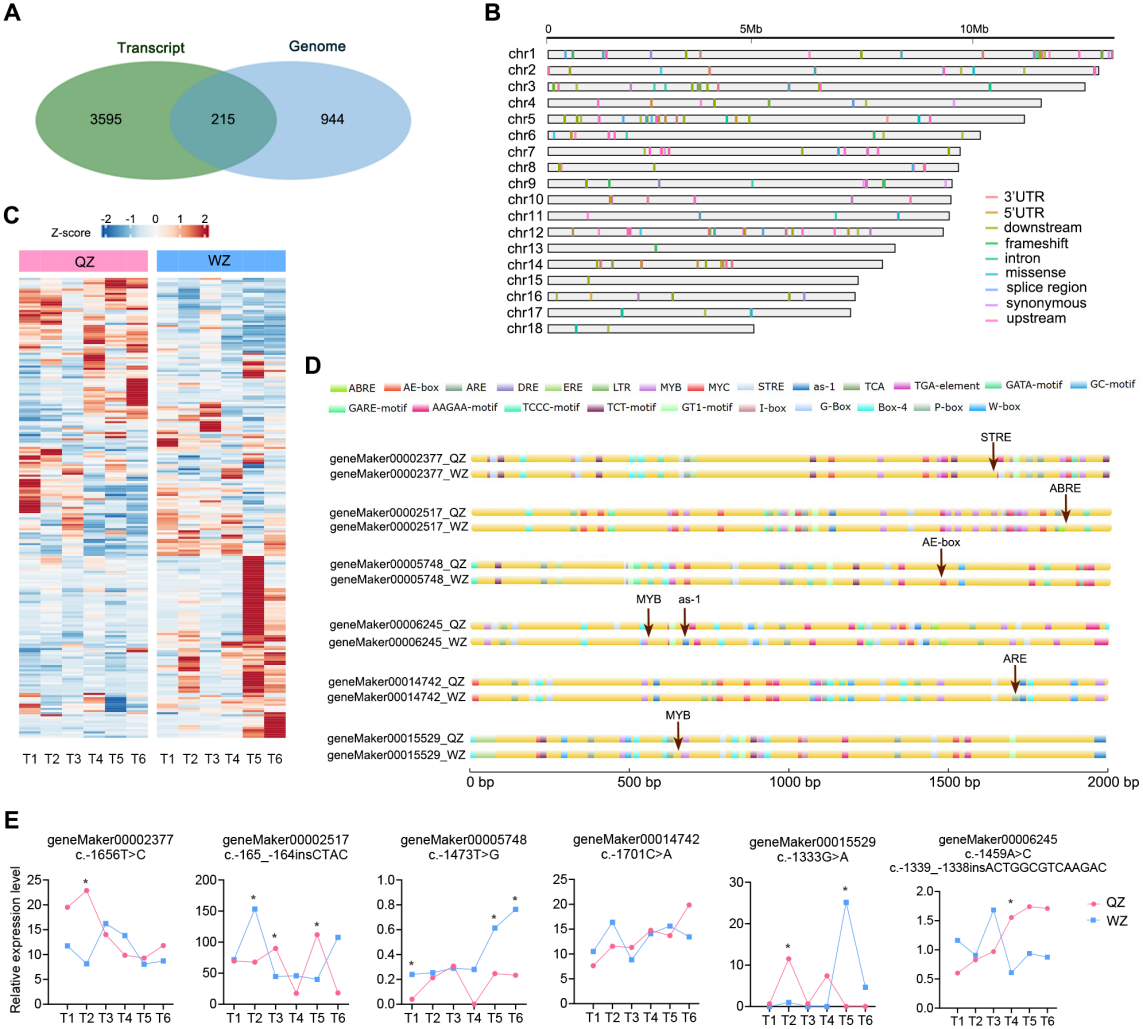
Integrated analysis of transcriptome and genome. **(A)** A Venn diagram illustrating the overlap of genes identified through *F*_ST_-based genomic positive selection analysis and transcriptome DEGs under cold wave, resulting in 215 genes. **(B)** Visualization of SNP variation types and their chromosomal locations within the 215 genes, with each row representing a distinct chromosome and different colors denoting various variation types. **(C)** A heatmap displaying the transcription level changes of the 215 genes under cold wave. **(D)** The impact of upstream base mutations on *cis-*elements of *K*. *obovata* genes in two populations, indicated by red arrows and text depicting *cis-*elements before and after mutations. **(E)** The differential transcription levels of selected genes in the cold wave (* *p*<0.05).

In our previous study, we examined the transcriptome profile of QZ and WZ during a simulated cold wave (**Supplementary Figure 8**), identifying 3,810 DEGs (Zhang et al., 2024). In this study, we conducted an analysis of 1,159 genes exhibiting positive selection, as identified from the *F*_ST_ index scan (within the top 5%) of the ZZ and WZ populations based on resequencing data. Upon taking the intersection of these gene sets, we obtained 215 genes (**Figures 4A, B**; **Supplementary Table 11**), which have shown differential transcription changes in the cold wave (**Figure 4C**). These genes were subsequently subjected to SNP mining and functional annotation analysis. Our findings revealed a total of 592 variants, including 206 variations within 1 kb region upstream of transcription start site (TSS), 195 variations within 1 kb region downstream of transcription end site, 96 variations within introns, as well as 39 missense mutations and 18 synonymous mutations (**Supplementary Table 12**).

The differential expression of genes can be attributed to the presence of distinct *cis*-acting elements within various gene promoters. Variations in upstream sequences have the potential to modify these *cis*-acting elements and binding sites for transcription factors, thereby influencing their binding capacity and efficiency, consequently impacting gene expression levels. Genes exhibiting variations in their upstream sequences were then screened, and promoter analysis was conducted for these varied upstream sequences. Substantial disparities in *cis*-regulatory elements between the WZ and QZ populations have been found. For instance, within geneMaker00002377, substitution of A for G at nucleotide position 1656 in the QZ population results in an additional stress response element (STRE) present exclusively in the WZ population. Similarly, within the geneMaker00002517 encoding drought-induced protein, there is an extra abscisic acid response element (ABRE) present solely in the WZ population compared to QZ; this element can be recognized by bZIP transcription factors leading to increased expression under cold conditions and thus enhancing cold tolerance of *K. obovata* (Sun et al., 2022). Furthermore, there is an elevated C allele frequency at nucleotide position 1473 within geneMaker00005748 in WZ population which encodes *K. obovata* homologue of E3 ligase SHOOT GRAVITROPISM9 (SGR9) (Nakamura et al., 2011), resulting in an additional AE-box response element which may enhance cold tolerance. Additionally, the substitution from G to A at nucleotide position 1333 within extracellular signal-regulated kinase (ERK) activator CEP14-encoding gene (geneMaker00015529) (Delay et al., 2013) leads to an inclusion of MYB element exclusively found in WZ that could potentially bolster plant recovery ability under low temperature stress during later stages (**Figures 4D, E**; **Table 4**). We propose that these differences may account for alterations in target gene transcription levels between the two populations and potentially contribute to changes in plant cold tolerance.

**Table 4:** Effects of upstream variation on *cis*-elements and functions of the *K. obovata.*.

Gene id	Orthologs in Arabidopsis(Gene symbol)	Gene description	Mutation	Mutated cis-Acting Element	TF binding Sequence	TF	Function of Site
geneMaker00002377	–	–	c.-1656T>C	STRE	AGGGG	–	Cis-acting elements involved in stress responsiveness
geneMaker00002517	–	drought-induced protein	c.-165_-164insCTAC	ABRE	ACGTG	bZIP	cis-acting element involved in the abscisic acid responsiveness
geneMaker00005748	SHGR9	E3 ubiquitin-protein ligase SGR9, amyloplastic [Manihot esculenta]	c.-1473T>G	AE-box	AGAAACAA	Trihelix	part of a module for light response
geneMaker00014742	RIPK	probable serine/threonine-protein kinase PBL12 [Ricinus communis]	c.-1701C>A	ARE	AAACCA	MYB recognition site	cis-acting regulatory element essential for the anaerobic induction
geneMaker00015529	PCP14	Precursor of CEP14	c.-1333G>A	MYB	TAACCA	MYB recognition site	MYB binding site
geneMaker00006245	PTL	trihelix transcription factor PTL-like [Hevea brasiliensis]	c.-1459A>C	MYB	TAACCA	MYB recognition site	MYB binding site
			c.-1339_-1338insACTGGCGTCAAGAC	as-1	TGACG	bZIP	cis-acting element involved in salicylic acid responsiveness

We also identified a significant number of mutations in the downstream gene sequences of DEGs. For example, the *CBF3* gene (geneMaker00008995), which constitutes the core of the cold stress pathway and plays an essential upstream initiation role in *K. obovata* cold resistance (Peng et al., 2020), exhibits an AA base deletion at locus 938050 on chromosome 9 in the WZ population. Furthermore, the *ABI5* gene (geneMaker00005324), a negative feedback factor in abscisic acid (ABA)-signaling involved in regulating ABA signaling pathway and ROS levels (Collin et al., 2021), shows a C to A base replacement at locus 2261919 on chromosome 5 within the WZ population. The potential relationship between these variants and changes in transcription levels of corresponding genes remains uncertain yet.

## 4 Discussion

### 4.1 Variation in phenotypic and leaf functional traits of *K. obovata* from different populations

Under the influence of natural and artificial selection, plants will inevitably differentiate in terms of phenotype and ecological traits as they adapt to new environments, gradually forming distinct geographical sources and leading to significant differences in morphology and functional leaf traits. Morphological indices such as leaf thickness, leaf dry matter content, leaf water content, and specific leaf area can reflect the plant’s adaptive features and ability to acquire resources in different habitats. Specific leaf area is typically closely linked to the plant’s growth strategies for survival. Plants with low specific leaf area often thrive in harsher or less fertile environments, while those with high specific leaf area can effectively maintain their nutrient content (Meziane and Shipley, 1999). Furthermore, as an index of ecological adaptation, leaf anatomical structure is closely related to a plant’s cold tolerance; it is frequently used as a key metric for evaluating plant cold tolerance. Generally speaking, the higher the ratio of palisade to mesophyll tissue density within leaves (and thus tighter structural density), the greater their cold tolerance becomes. In response to adverse conditions such as high temperatures, small dense stomata are advantageous for avoiding rapid water loss due to transpiration.

QZ and WZ populations have been introduced into Fujian Quanzhou and Zhejiang Wenzhou within the last 20 years. Despite this study’s elimination of native habitat effects through a CGE, these populations have already displayed distinctive phenotypic variations and leaf traits. The research findings suggest that the QZ population at lower latitudes exhibits greater PH, BD, W, LN and BN. Moreover, there are notable disparities in leaf functional traits and anatomical structures between the two populations. The WZ population demonstrates a larger SLA, potentially attributed to its robust photosynthetic capacity facilitating rapid resource acquisition and internal nutrient maintenance. Its palisade tissue is thicker with a denser cell structure, resulting in a higher P/S that enhances cold resistance. Conversely, the QZ population features smaller SA and higher SD possibly linked to relatively elevated local temperatures and strong transpiration for favorable water balance. In contrast, the WZ population displays larger but sparser stomata which may enhance its resilience to adverse conditions by reducing transpiration rate (**Figures 1A, B**).

It is widely accepted that there exists a trade-off between plant growth and defense. When plants are frequently exposed to pathogens or adverse environmental conditions, they prioritize defense mechanisms, leading to a reduction in growth and reproductive capacity (Zhou et al., 2022). We posit that the seedlings of WZ adopted a distinct energy allocation strategy compared to those of QZ in order to more effectively acclimate to the high-latitude winter environment. Additionally, the functional characteristics of the leaves underwent changes aimed at enhancing their adaptability to the environment, with various leaf indices demonstrating a synergistic and trade-off relationship.

Consequently, we postulate that the WZ population of *K. obovata* inhabiting relatively cold environments may exhibit adaptive responses by modifying its growth-defense trade-off strategy. This could involve allocating more resources to defense mechanisms, slowing down the growth rate, increasing leaf tissue density and structural compactness, reducing SLA, and regulating stomatal characteristics as an adaptive response to the challenging habitat. These adaptive adjustments are likely to enhance the plant’s resistance to low temperatures and improve its environmental adaptability and resource utilization. Conversely, the QZ population in milder habitats may allocate greater energy towards growth and reproduction. In conclusion, this study unveils significant phenotypic disparities between WZ and QZ, suggesting the adaptive capacity of *K. obovata* to environmental fluctuations. The heterogeneity of the environment over the approximately 20-year adaptation period may underlie the observed differentiation in phenotypic traits, leaf functionality, and physiological characteristics between QZ and WZ populations.

### 4.2 Genetic diversity and kinship analysis of different populations in the same introduction

The research conducted by Yang et al. demonstrates a substantial variation in both hypocotyl and seedling growth traits of *K. obovata* across diverse collection sites, showing a strong correlation with geographical and climatic factors (Yang et al., 2020). Prolonged acclimatization to its indigenous habitat can result in genetic divergence concerning plant morphology and leaf functional traits, thereby ensuring stable inheritance of these variations across successive generations. Our findings demonstrate that the mangrove species *K. obovata*, which was introduced from ZZ to WZ and QZ within a brief two-decade period, rapidly acclimated to the local environment through the development of specific traits. Significant phenotypic differences were already evident in the hypocotyls (**Figure 1**; **Supplementary Figure 1**).

The CGE further confirms that *K. obovata* seedlings also demonstrate significant variability in phenotype, leaf functional traits, and cold tolerance between the QZ and WZ populations (**Figure 1**, **Table 1**). The results of PCA and phylogenetic tree analysis based on WGRS indicate that ZZ and QZ are intertwined and clustered into the same branch, while WZ forms a separate cluster (**Figures 2A, B**). Additionally, there exists moderate genetic differentiation between the WZ and QZ populations, with experimental findings consistent with the geographic distribution of *K. obovata*. These results suggest that the observed phenotypic differences between the two *K. obovata* populations are primarily attributed to genetic differentiation rather than phenotypic plasticity.

This study utilized WGRS technology to assess the genetic diversity levels in various *K. obovata* populations. The analysis involved calculating heterozygosity (He, Ho), nucleotide diversity, polymorphic information content, and Shannon index. The findings revealed a decrease in genetic diversity of *K. obovata* populations in China with increasing latitude. At the whole-genome level, DZG exhibited the highest genetic diversity at lower latitudes, while ZZ and QZ displayed similar levels of genetic diversity and WZ showed the lowest (**Table 2**). Furthermore, observed heterozygosity (Ho) for ZZ, QZ, and WZ populations exceeded expected heterozygosity (He) (**Table 2**), suggesting higher frequency fluctuations possibly due to founder effect and bottleneck effect during the northward expansion of *K. obovata* driven by human selection of breeding individuals. The initial genetic bottleneck during the establishment of the WZ population likely resulted in limited genetic variation, leading to random phenotypic changes, some of which may be attributed to the founder effect. However, as the WZ population adapted to low-temperature environment, natural selection likely accelerated the optimization of these variants, driving the positive selection of cold-tolerance-related genes. For instance, we identified significant enrichment in pathways such as “related to stress response,” and “glutathione metabolism” (**Figure 3D**), along with selection signals linked to ABA/JA and low-temperature response elements, which are crucial for cold stress adaptation (**Figure 3E**). The WZ population has developed robust cold tolerance under prolonged exposure to low temperatures, and this trait has remained genetically stable across multiple generations, as confirmed by CGE in Shanghai (**Table 1**; **Figure 1**). The WZ population consistently exhibited enhanced overwinter survival rates (**Table 1**) and stronger cold-resistant phenotypes, accompanied by adaptive leaf anatomical changes, including thicker palisade tissue and a higher palisade-to-spongy ratio (**Figure 1**). These traits showed consistent inheritance, supporting the role of adaptive evolution in shaping this phenotype. Thus, while the founder effect may have initially constrained genetic diversity in the WZ population, the enhanced cold tolerance is more likely the result of adaptive evolution driven by natural selection. In conclusion, the phenotypic changes observed in the WZ population reflect a combination of adaptive evolution and the founder effect. Further studies, such as whole-genome association studies (GWAS) or quantitative trait locus (QTL) analysis, are needed to explore other phenotypic differences beyond cold tolerance.

Transcription factors regulate the expression of a wide range of biological and abiotic stress response genes by interacting with *cis*-acting elements in the promoter region, enabling plants to adapt to diverse stresses. To further explore potential stress response elements in the promoters of robust WZ populations, we utilized the Plantcare software for cis-element prediction in their promoter regions. The analysis revealed a substantial presence of cis-elements associated with abiotic stress response, such as LTR and hormone response elements (**Figure 3E**), suggesting their significant role in plant growth, development, low-temperature stress responses, and modulation of hormone signaling molecules. Endogenous plant hormones play a pivotal role in abiotic stress (Waadt et al., 2022). This investigation identified ABRE within almost all positively selected genes. Furthermore, most genes contain jasmonic acid (JA) response elements CGTCA-motif and TGACG-motif within their promoter sequences which facilitate ABA- and JA-dependent signaling pathways through interaction with upstream transcription factors. ABA and JA, essential endogenous plant hormones, are crucial in mediating abiotic stress responses and have been shown to significantly impact on low temperature stress in plants (Huang et al., 2017; Hu et al., 2017). Studies have shown that exogenous ABA application can significantly improve cold tolerance in many plants, such as *Capsicum annuum* (Guo et al., 2012) and *Vitis vinifera* (Wang et al., 2020). Similarly, JA positively regulates the ICE-CBF pathway to enhance cold tolerance in *Arabidopsis thaliana*, and exogenous application of methyl jasmonate (MeJA) significantly improves cold tolerance in plants (Hu et al., 2013, Hu et al., 2017). Notably, exogenous ABA can alleviate cold stress in *K. obovata* by activating antioxidant enzyme activities and promoting the accumulation of osmotic regulators, thereby mitigating the negative effects of cold stress (Liu et al., 2022). Furthermore, in our previous research, we found that the enhanced cold tolerance in the WZ population is linked to JA signaling molecules and exogenous application of MeJA reduced cold-induced damage in the QZ population (Zhang et al., 2024). These findings suggest that these genes may be regulated by ABA and JA pathways thereby promoting *K. obovata*’s response to low temperature stress.

### 4.3 Low Temperature Maybe a Major Factor in the Genetic Differentiation of *K. obovata*

Climate exerts significant selective pressure on plant traits, with low temperature playing a crucial role in influencing the distribution of mangrove plants at high latitudes. Previous studies have shown that temperature is a major environmental factor limiting the distribution of mangroves (Cavanaugh et al., 2014). For instance, extreme freezing events are considered as a primary environmental factor determining the latitudinal distribution of *K. obovata* (Su et al., 2019; He et al., 2023) or other species such as *Avicennia germinans* (Pickens and Hester, 2011; Osland et al., 2014). A related study integrated geographical distribution data for *K. obovata* from both natural and introduced populations along China’s southeastern coast and combined it with climate and hydrological data to quantitatively analyze the relationship between geographical distribution patterns and key environmental factors (Zhu et al., 2021). The results indicated that the top five climate factors influencing *K. obovata*’s distribution were annual mean temperature, mean temperature of the coldest quarter, extreme minimum temperature, temperature seasonality, and mean temperature of the driest quarter. These findings are consistent with our observations. On the one hand, phenotypic and genomic analyses revealed temperature-associated variations, including selection signatures at cold-responsive elements and promoter polymorphisms in cold adaptation genes (**Table 1**; **Figures 3**, **4**). On the other hand, based on data from the China Meteorological Science Data Sharing Service Network (http://data.cma.cn), the average temperature and lowest temperature of the coldest month in the WZ and QZ regions over the past 75 years are as follows: 8.1°C and 12.0°C for the average temperatures of the coldest month in the WZ and QZ regions respectively; -0.9°C and 5.5°C for their respective average extreme low temperatures in the coldest month (**Supplementary Figure 9**; **Supplementary Table 13**). These findings indicate that temperature likely serves as a primary driver of genetic differentiation between populations of *K. obovata* introduced from related source sites (WZ and QZ) due to substantial differences in latitude, average temperature, and other environmental conditions. However, it should be noted that while temperature gradients strongly correlate with latitudinal variation, other environmental parameters may synergistically shape genetic differentiation. Although WZ is located at a higher latitude and experiences colder temperatures, the observed genetic differentiation may result from a complex interplay of multiple environmental pressures, rather than temperature alone. Future studies that collect additional long-term environmental data—such as salinity, precipitation, and soil parameters—from both QZ and WZ will provide a more comprehensive analysis of the multi-layered environmental factors influencing the genetic structure and distribution of *K. obovata*.

Following transplantation to higher latitudes, acclimatization processes occurred within the ZZ population, leading to elimination of mangrove plants ill-suited for low temperatures while retaining those adapted to such conditions, ultimately giving rise to today’s WZ population which demonstrates enhanced adaptability to low temperatures at high latitudes (**Table 1**). The molecular mechanisms underlying adaptation to low temperatures by the WZ population have been partially elucidated (Su et al., 2019). In summary, both natural selection and genetic drift play crucial roles in shaping the adaptation of *K. obovata* to its environment. After approximately 20 years, phenotypic and nucleotide-level genetic differentiation between the WZ and ZZ populations indicates that *K. obovata* possesses potential for rapid adaptation, rendering it a promising candidate species for studying plant adaptability.

## 5 Conclusion

In conclusion, this study investigated the phenotypic and functional trait variations of WZ and QZ populations in common garden experience. The findings revealed that different populations exhibited distinct growth-defense trade-off strategies to adapt to their respective environments. Within the same introduction site, moderate genetic differentiation was observed among *K. obovata* populations, indicating the rapid adaptation ability of *K. obovata* to environmental changes. WGRS further elucidated the genetic structure of *K. obovata* populations within the same introduction site, with results showing that the northernmost artificially introduced population (WZ) displayed the lowest genetic diversity. Additionally, its gene promoter regions affected by strong selection contained a significant number of stress response elements related to low temperature and hormones, suggesting that temperature may be a primary driver of genetic differentiation in *K. obovata*. These research findings contribute to our understanding of environmental adaptation characteristics and growth/survival strategies of *K. obovata* in introduction areas, providing theoretical support for its cultivation as well as for mangrove forest protection and development efforts.

## Data Availability

The WGRS data presented in the study are deposited in the NCBI repository, accession number PRJNA1145277 (https://www.ncbi.nlm.nih.gov/sra/PRJNA1145277). The transcriptome data cited in this study are deposited in the NCBI repository, accession number PRJNA1093421 (https://www.ncbi.nlm.nih.gov/sra/PRJNA1093421).
