# Supplementary material for: Phenotypic adaptation and genomic variation of Kandelia obovata associated with its northern introduction along southeastern coast of China
Source: Front Plant Sci. 2025 Mar 26;16:1512620. doi: 10.3389/fpls.2025.1512620 (PMC11980879; doi:10.3389/fpls.2025.1512620)
Supplement: Supplementary file 2 [file DataSheet1.docx]

**Supplementary material**


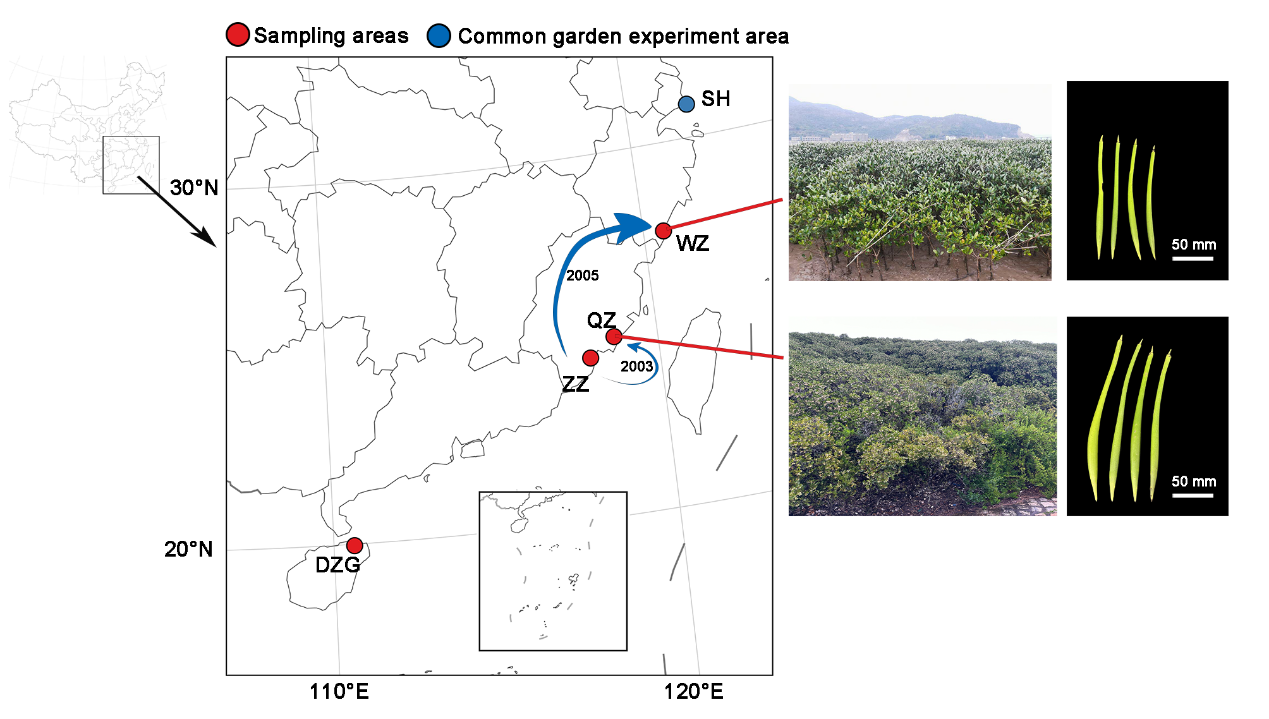


**Supplementary Figure 1.** Plant materials for CGE and/or WGRS.

(**Left panel**) The distribution map of the mangrove samples utilized in this study is presented, with the horizontal axis representing longitude and the vertical axis representing latitude. Red circles indicate sampling sites for WGRS, while blue circles represent sampling sites for CGE. Blue arrows depict the history and timeline of mangrove introductions. **DZG** refers to Dongzhaigang Mangrove Nature Reserve in Haikou City, Hainan Province (110°58'E,19°95'N); **ZZ** represents Jiulong River Estuary Mangrove Provincial Nature Reserve in Zhangzhou City, Fujian Province (117°92'E,24°46 N); **QZ** denotes Luoyang River Mangrove Nature Reserve in Quanzhou Bay, Quanzhou City, Fujian Province (118°59'E,24°91'N); **WZ** stands for Longgang Aojiang Estuary Mangrove in Wenzhou City, Zhejiang Province (120°96'E,28°12'N). (**Right panel**) Introduced *K. obovata* in QZ and WZ (left panel) and the comparison of their hypocotyls, bar=50 mm.

**
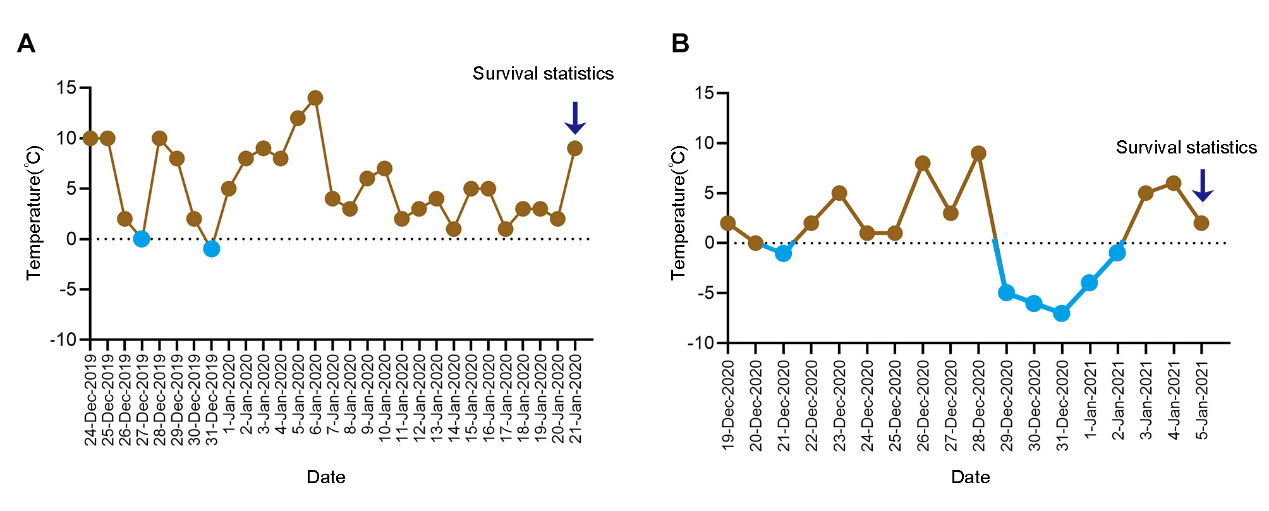
**

**Supplementary Figure 2.** The daily minimum temperature changes at Nanhui Dongtan Mangrove Base during the coldest months of 2020 and 2021.

**
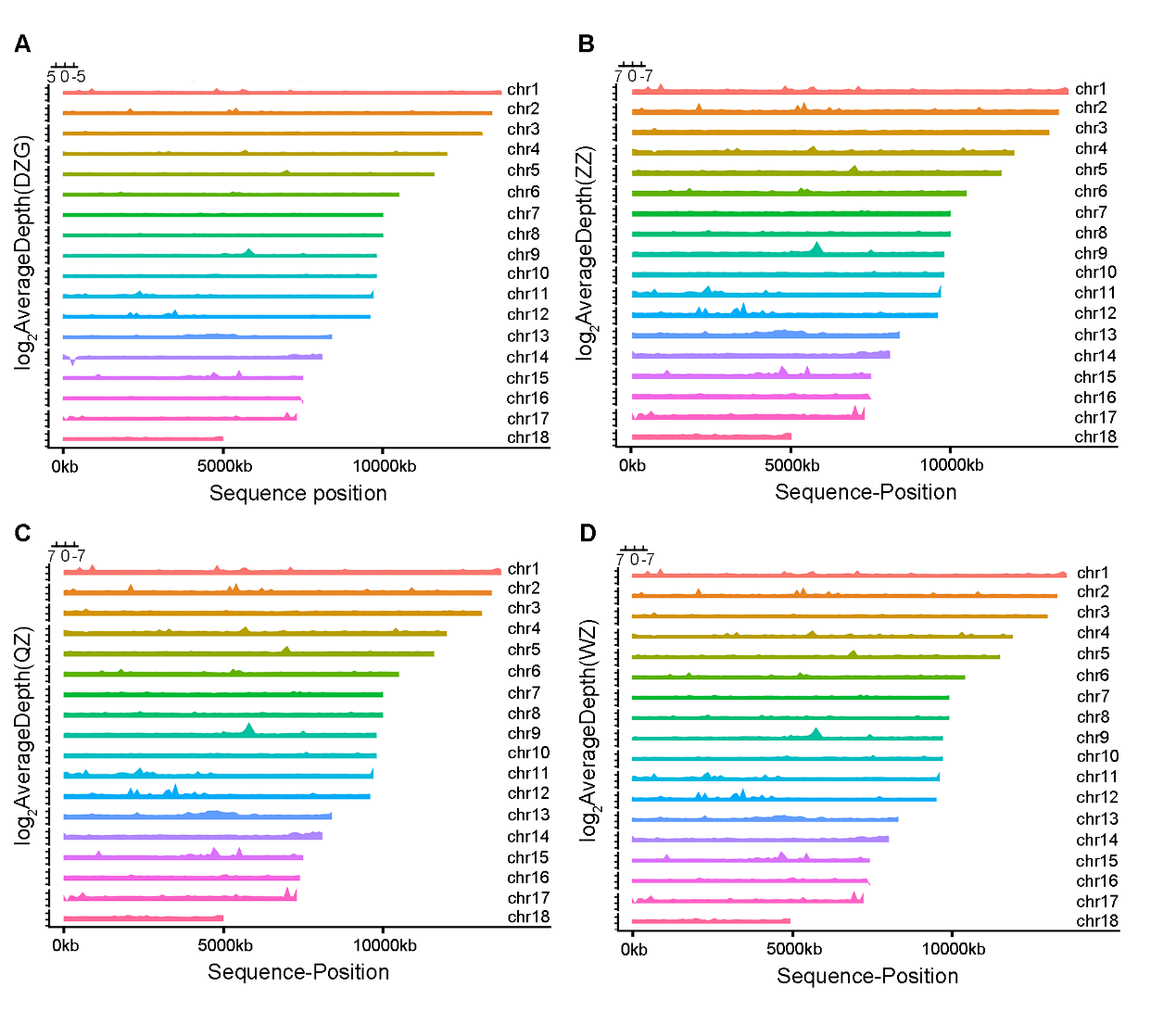
**

**Supplementary Figure 3.** Histogram depicting the distribution of chromosome coverage depth among different populations.

(**A**) DZG population; (**B**) ZZ population; (**C**) QZ population; and (**D**) WZ population. The horizontal axis represents the chromosomal positions, while the vertical axis denotes the log_2_-transformed coverage depth at each respective position on the chromosome.

**
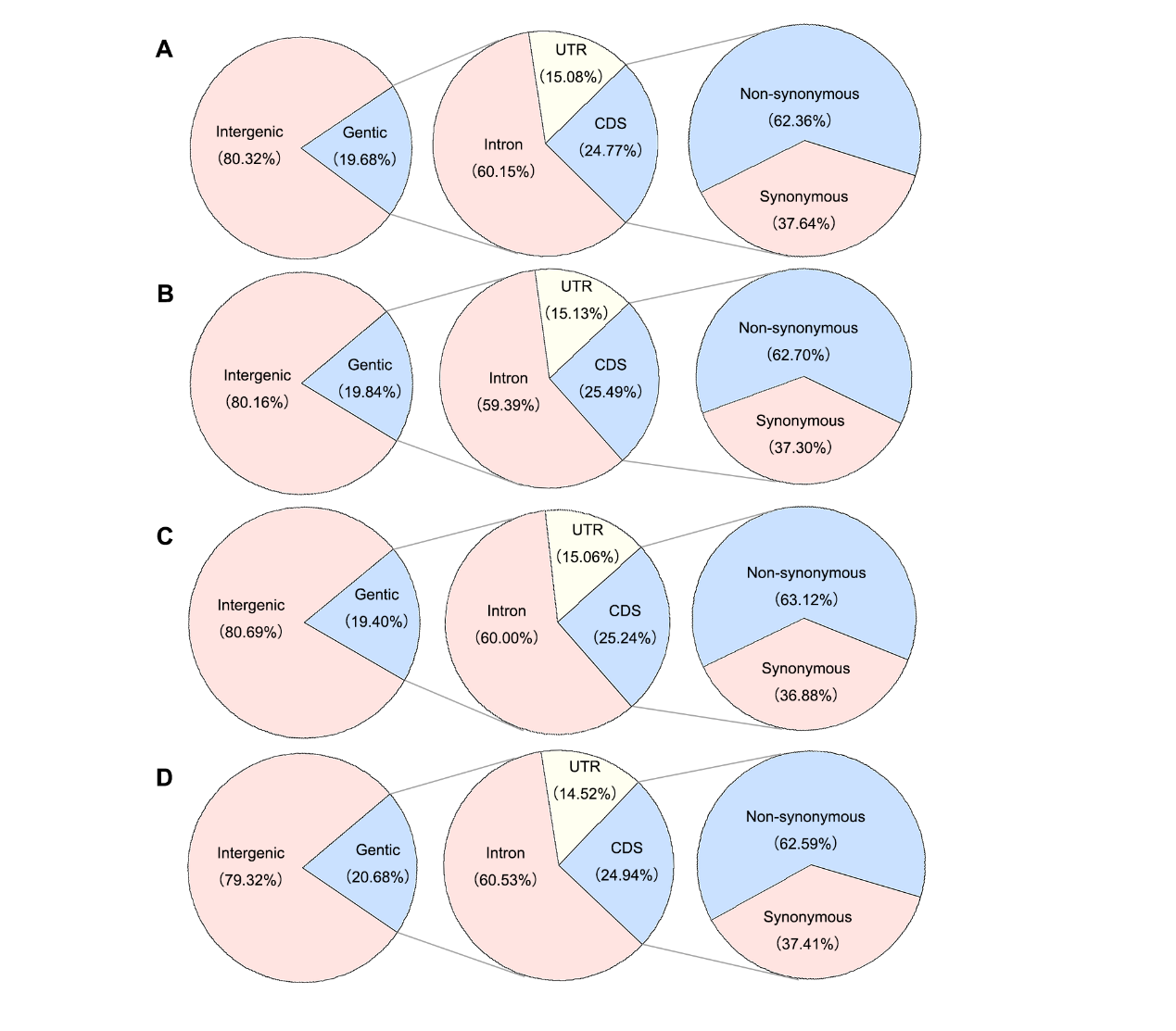
**

**Supplementary Figure 4.** The SNP annotation results on chromosomes for various *K. obovata* populations.

(A) DZG population; (B) ZZ population; (C) QZ population; and (D) WZ population.

**
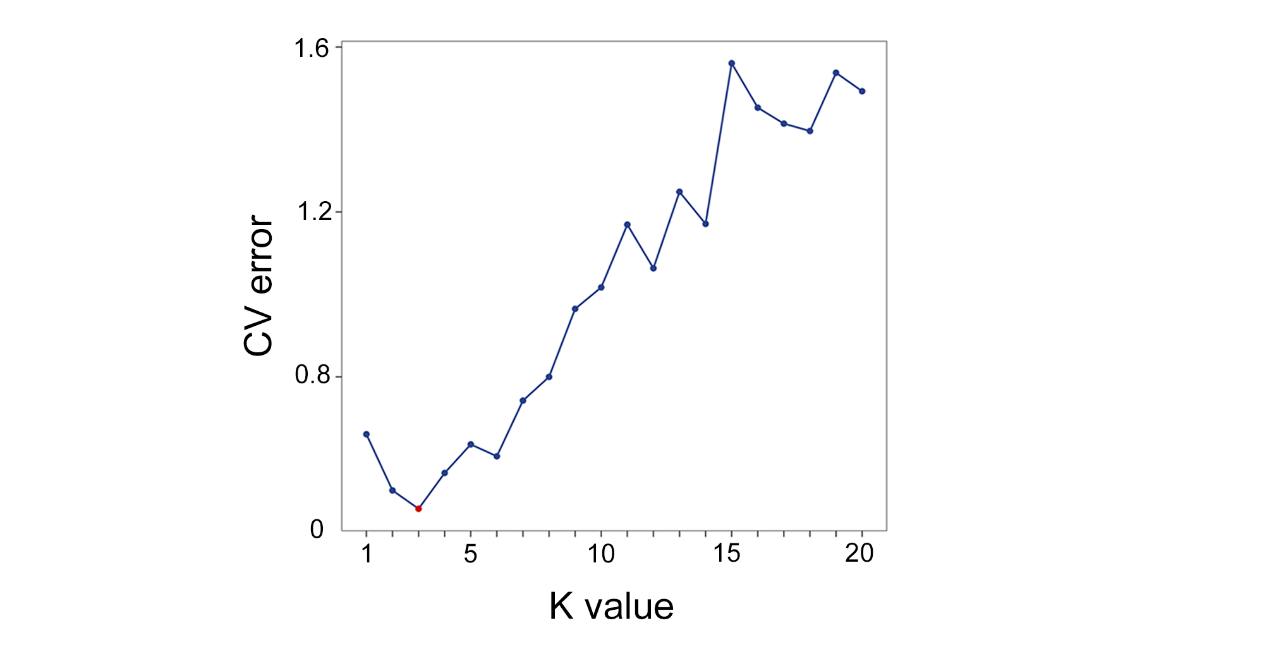
**

**Supplementary Figure 5.** Cross-validation error rate for K values.

The horizontal axis represents different K values, and the vertical axis represents the variation error rate. The red circle indicates the point with the lowest variation error rate.


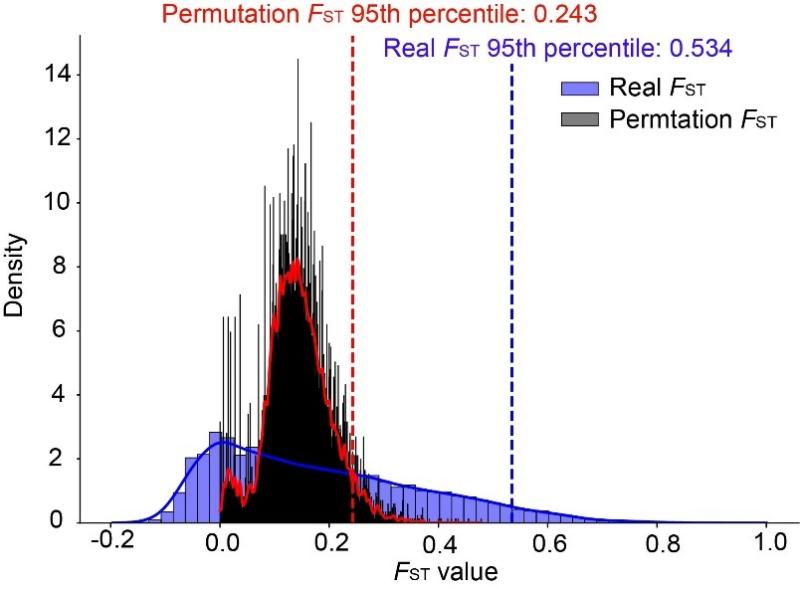


**Supplementary Figure 6. Comparison of 𝐹_ST_ distribution after 1,000 permutation tests.**The red dashed line represents the 95^th^ percentile threshold derived from the permutation test, while the blue dashed line represents the real 95^th^ percentile threshold for 𝐹_ST_ in the population.


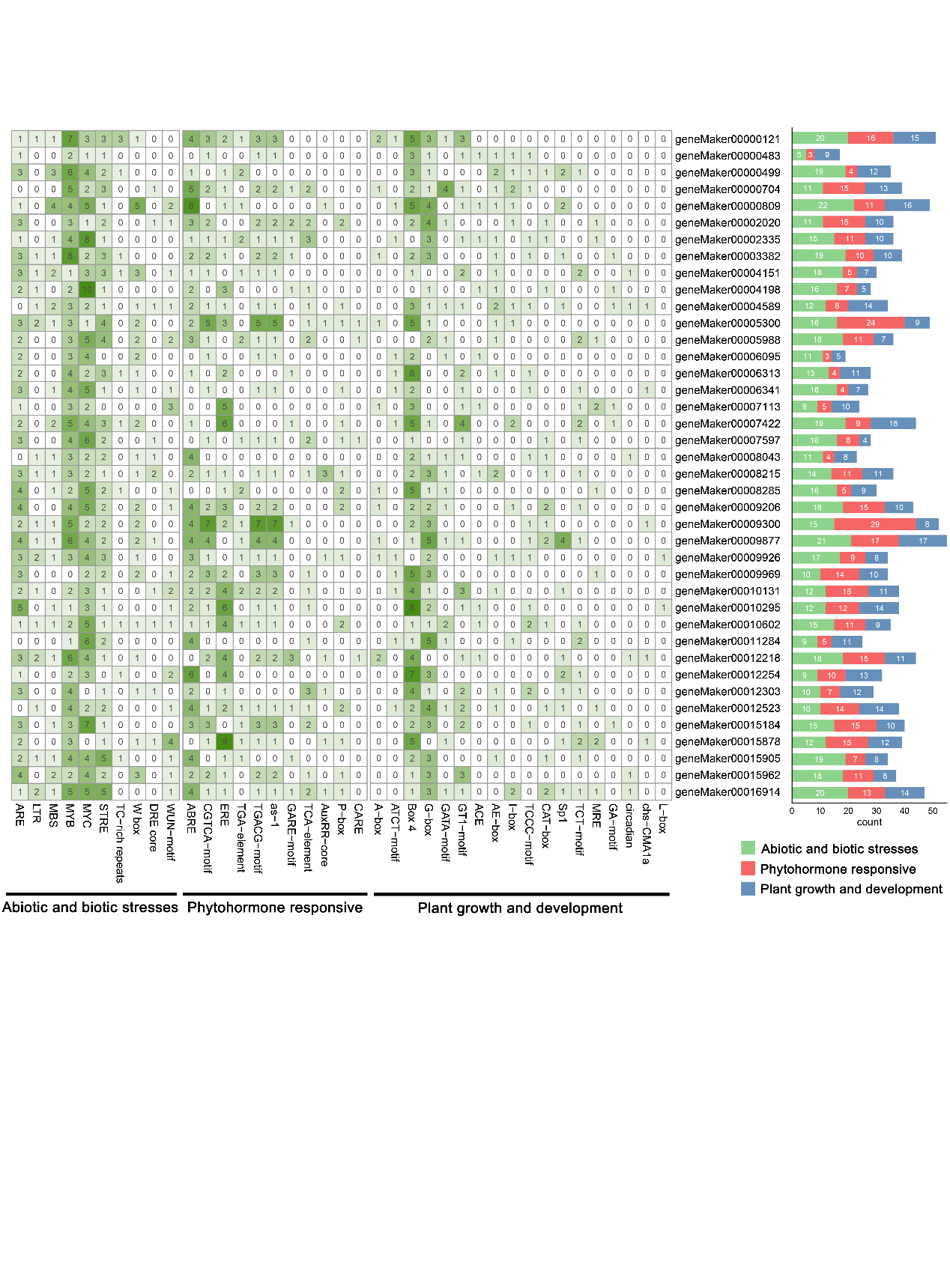


**Supplementary Figure 7.** Prediction of *cis*-acting elements in the promoter region of all 40 WZ selected genes.

The *cis*-acting elements were classified into three categories according to their functions, with green indicating abiotic and biotic stresses, red indicating phytohormone responsive, and blue indicating plant growth and development. The numbers in the squares on the left represent the number of cis-elements per gene, and the numbers on the right represent the number of *cis*-elements with different functions per gene.


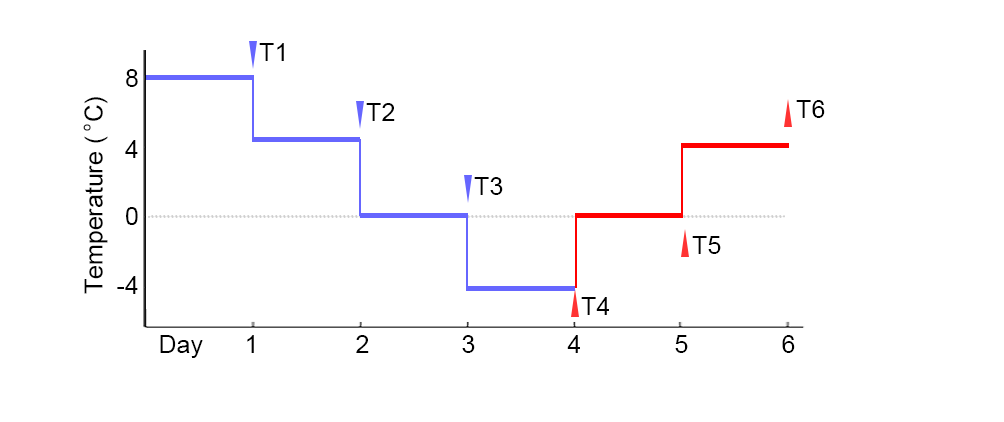


**Supplementary Figure 8.** Simulated cold wave in our previous study (Zhang et al., 2024).

Experimental design employed to assess the phenotype at the end of each temperature treatment. The cold wave involved exposure to temperature of 8℃ for 1 day (T1), followed by 4℃ for 1 day (T2), then 0℃ for 1 day (T3), and finally -4℃ for 1 day (T4). Subsequently, rewarming treatment was conducted at temperature of 0℃ for 1 day (T5), followed by 4℃ for 1 day (T6).


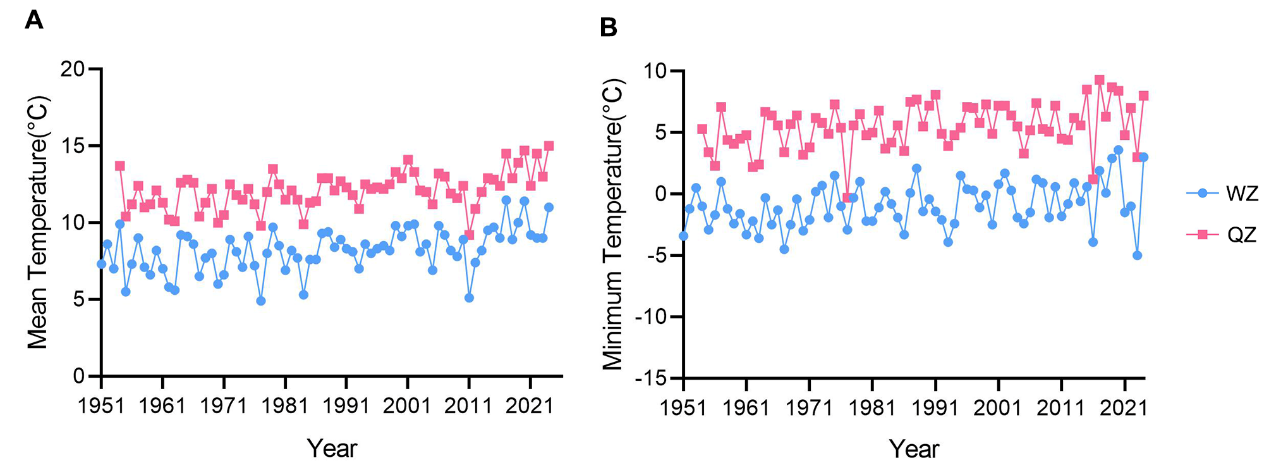


**Supplementary Figure 9.** Change of the mean and minimum temperature of the coldest month in WZ and QZ during 1951-2024.

(**a**) Change in the mean temperature of the coldest month; (b) Change of minimum temperature in the coldest month. Blue represents the WZ region and red represents the QZ region.

**References**

Alexander, D.H., Novembre, J., and Lange, K. (2009). Fast model-based estimation of ancestry in unrelated individuals. *Genome Res* 19**,** 1655-1664. doi: 10.1101/gr.094052.109

Bastias, C.C., Estarague, A., Vile, D., Gaignon, E., Lee, C.R., Exposito-Alonso, M., Violle, C., and Vasseur, F. (2024). Ecological trade-offs drive phenotypic and genetic differentiation of Arabidopsis thaliana in Europe. *Nat Commun* 15**,** 5185. doi: 10.1038/s41467-024-49267-0

Baythavong, B.S., and Stanton, M.L. (2010). Characterizing selection on phenotypic plasticity in response to natural environmental heterogeneity. *Evolution* 64**,** 2904-2920. doi: 10.1111/j.1558-5646.2010.01057.x

Bjorkman, A.D., Vellend, M., Frei, E.R., and Henry, G.H. (2017). Climate adaptation is not enough: warming does not facilitate success of southern tundra plant populations in the high Arctic. *Glob Chang Biol* 23**,** 1540-1551. doi: 10.1111/gcb.13417

Boquete, M.T., Muyle, A., and Alonso, C. (2021). Plant epigenetics: phenotypic and functional diversity beyond the DNA sequence. *Am J Bot* 108**,** 553-558. doi: 10.1002/ajb2.1645

Catchen, J., Hohenlohe, P.A., Bassham, S., Amores, A., and Cresko, W.A. (2013). Stacks: an analysis tool set for population genomics. *Mol Ecol* 22**,** 3124-3140. doi: 10.1111/mec.12354

Cavanaugh KC, Kellner JR, Forde AJ, Gruner DS, Parker JD, Rodriguez W, Feller IC. (2014). Poleward expansion of mangroves is a threshold response to decreased frequency of extreme cold events. *Proc Natl Acad Sci U S A* 2, 723-727. doi: 10.1073/pnas.1315800111

Chen, C., Chen, H., Zhang, Y., Thomas, H.R., Frank, M.H., He, Y., and Xia, R. (2020). TBtools: An Integrative Toolkit Developed for Interactive Analyses of Big Biological Data. *Mol Plant* 13**,** 1194-1202. doi: 10.1016/j.molp.2020.06.009

Chen, L., Wang, W., Li, Q.Q., Zhang, Y., Yang, S., Osland, M.J., Huang, J., and Peng, C. (2017). Mangrove species' responses to winter air temperature extremes in China. 8**,** e01865. doi: 10.1002/ecs2.1865.

Chen, S., Zhou, Y., Chen, Y., and Gu, J. (2018). fastp: an ultra-fast all-in-one FASTQ preprocessor. *Bioinformatics* 34**,** i884-i890. doi: 10.1093/bioinformatics/bty560

Cingolani, P., Platts, A., Wang Le, L., Coon, M., Nguyen, T., Wang, L., Land, S.J., Lu, X., and Ruden, D.M. (2012). A program for annotating and predicting the effects of single nucleotide polymorphisms, SnpEff: SNPs in the genome of Drosophila melanogaster strain w1118; iso-2; iso-3. *Fly (Austin)* 6**,** 80-92. doi: 10.4161/fly.19695

Collin, A., Daszkowska-Golec, A., and Szarejko, I. (2021). Updates on the Role of ABSCISIC ACID INSENSITIVE 5 (ABI5) and ABSCISIC ACID-RESPONSIVE ELEMENT BINDING FACTORs (ABFs) in ABA Signaling in Different Developmental Stages in Plants. *Cells* 10. doi: 10.3390/cells10081996

Guo WL, Chen RG, Gong ZH, Yin YX, Ahmed SS, He YM. (2012). Exogenous abscisic acid increases antioxidant enzymes and related gene expression in pepper (*Capsicum annuum*) leaves subjected to chilling stress. *Genet Mol Res* 11, 4063-4080. doi: 10.4238/2012.September.10.5

Danecek, P., Auton, A., Abecasis, G., Albers, C.A., Banks, E., Depristo, M.A., Handsaker, R.E., Lunter, G., Marth, G.T., Sherry, S.T., Mcvean, G., and Durbin, R. (2011). The variant call format and VCFtools. *Bioinformatics* 27**,** 2156-2158. doi: 10.1093/bioinformatics/btr330

Delay, C., Imin, N., and Djordjevic, M.A. (2013). CEP genes regulate root and shoot development in response to environmental cues and are specific to seed plants. *J Exp Bot* 64**,** 5383-5394. doi: 10.1093/jxb/ert332

Ellison, A.M., Felson, A.J., and Friess, D.A. (2020). Mangrove Rehabilitation and Restoration as Experimental Adaptive Management. *Frontiers in Marine Science* 7**,** 19. doi: 10.3389/fmars.2020.00327

Foyer, C.H., and Kranner, I. (2023). Plant adaptation to climate change. *Biochem J* 480**,** 1865-1869. doi: 10.1042/bcj20220580

Freed, D., Aldana, R., Weber, J., and Edwards, J. (2017).The Sentieon Genomics Tools - A fast and accurate solution to variant calling from next-generation sequence data. doi: 10.1101/115717

He S, Wang X, Du Z, Liang P, Zhong Y, Wang L, Zhang YY, Shen Y. (2023). Physiological and transcriptomic responses to cold waves of the most cold-tolerant mangrove, *Kandelia obovata*. *Front Plant Sci* 14, 1069055. doi: 10.3389/fpls.2023.1069055

Hu, M.J., Sun, W.H., Tsai, W.C., Xiang, S., Lai, X.K., Chen, D.Q., Liu, X.D., Wang, Y.F., Le, Y.X., Chen, S.M., Zhang, D.Y., Yu, X., Hu, W.Q., Zhou, Z., Chen, Y.Q., Zou, S.Q., and Liu, Z.J. (2020). Chromosome-scale assembly of the Kandelia obovata genome. *Hortic Res* 7**,** 75. doi: 10.1038/s41438-020-0300-x

Hu Y, Jiang L, Wang F, Yu D. (2013). Jasmonate regulates the inducer of cbf expression-C-repeat binding factor/DRE binding factor1 cascade and freezing tolerance in Arabidopsis. *Plant Cell* 25, 2907-2924. doi: 10.1105/tpc.113.112631

Hu Y, Jiang Y, Han X, Wang H, Pan J, Yu D. (2017). Jasmonate regulates leaf senescence and tolerance to cold stress: crosstalk with other phytohormones. *J Exp Bot* 68, 1361-1369. doi: 10.1093/jxb/erx004

Huang X, Shi H, Hu Z, Liu A, Amombo E, Chen L, Fu J. (2017). ABA Is Involved in Regulation of Cold Stress Response in Bermudagrass. *Front Plant Sci* 8, 1613. doi: 10.3389/fpls.2017.01613

Jung, Y., and Han, D. (2022). BWA-MEME: BWA-MEM emulated with a machine learning approach. *Bioinformatics* 38**,** 2404-2413. doi: 10.1093/bioinformatics/btac137

Korunes, K.L., and Samuk, K. (2021). pixy: Unbiased estimation of nucleotide diversity and divergence in the presence of missing data. *Mol Ecol Resour* 21**,** 1359-1368. doi: 10.1111/1755-0998.13326

Lescot, M., Déhais, P., Thijs, G., Marchal, K., Moreau, Y., Van De Peer, Y., Rouzé, P., and Rombauts, S. (2002). PlantCARE, a database of plant cis-acting regulatory elements and a portal to tools for in silico analysis of promoter sequences. *Nucleic Acids Res* 30**,** 325-327. doi: 10.1093/nar/30.1.325

Li, C., Wang, F., Yang, P., Wang, F.C., Hu, Y.Z., Zhao, Y.L., Tian, L.Z., and Zhao, R.B. (2024). Mangrove wetlands distribution status identification, changing trend analyzation and carbon storage assessment of China. *China Geology* 7**,** 1-11. doi: 10.31035/cg2023049

Li, H., Handsaker, B., Wysoker, A., Fennell, T., Ruan, J., Homer, N., Marth, G., Abecasis, G., Durbin, R., and Genome Project Data Processing, S. (2009). The Sequence Alignment/Map format and SAMtools. *Bioinformatics* 25**,** 2078-2079. doi: 10.1093/bioinformatics/btp352

Liu X, Lu X, Yang S, Liu Y, Wang W, Wei X, Ji H, Zhang B, Xin W, Wen J, Wang J, Chen Q. (2022). Role of exogenous abscisic acid in freezing tolerance of mangrove *Kandelia obovata* under natural frost condition at near 32^°^N. *BMC Plant Biol* 22, 593. doi: 10.1186/s12870-022-03990-2

Lu, W.X., Zhang, B.H., and Yang, S.C. (2023). Survive the north: transplantation for conservation of mangrove forests requires consideration of influences of low temperature, mating system and their joint effects on effective size of the reforested populations. *Frontiers in Ecology and Evolution* 11**,** 10. doi: 10.3389/fevo.2023.1160468.

Lu, W.X., Zhang, B.H., Zhang, Y.Y., and Yang, S.C. (2021). Differentiation of Cold Tolerance in an Artificial Population of a Mangrove Species, Kandelia obovata, Is Associated With Geographic Origins. *Front Plant Sci* 12**,** 695746. doi: 10.3389/fpls.2021.695746

Marchini, G.L., Maraist, C.A., and Cruzan, M.B. (2019). Trait divergence, not plasticity, determines the success of a newly invasive plant. *Ann Bot* 123**,** 667-679. doi: 10.1093/aob/mcy200

Mckenna, A., Hanna, M., Banks, E., Sivachenko, A., Cibulskis, K., Kernytsky, A., Garimella, K., Altshuler, D., Gabriel, S., Daly, M., and Depristo, M.A. (2010). The Genome Analysis Toolkit: a MapReduce framework for analyzing next-generation DNA sequencing data. *Genome Res* 20**,** 1297-1303. doi: 10.1101/gr.107524.110

Meziane, D., and Shipley, B. (1999). Interacting determinants of specific leaf area in 22 herbaceous species: effects of irradiance and nutrient availability. *Plant Cell and Environment* 22**,** 447-459. doi: 10.1046/j.1365-3040.1999.00423.x

Minh, B.Q., Schmidt, H.A., Chernomor, O., Schrempf, D., Woodhams, M.D., Von Haeseler, A., and Lanfear, R. (2020). IQ-TREE 2: New Models and Efficient Methods for Phylogenetic Inference in the Genomic Era. *Mol Biol Evol* 37**,** 1530-1534. doi: 10.1093/molbev/msaa015

Miryeganeh, M. (2022). Mangrove Forests: Natural Laboratories for Studying Epigenetic and Climate Changes. *Front Plant Sci* 13**,** 851518. doi: 10.3389/fpls.2022.851518

Nakamura, M., Toyota, M., Tasaka, M., and Morita, M.T. (2011). An Arabidopsis E3 ligase, SHOOT GRAVITROPISM9, modulates the interaction between statoliths and F-actin in gravity sensing. *Plant Cell* 23**,** 1830-1848. doi: 10.1105/tpc.110.079442

Osland MJ, Day RH, Larriviere JC, From AS. (2014). Aboveground allometric models for freeze-affected black mangroves (*Avicennia germinans*): equations for a climate sensitive mangrove-marsh ecotone. *PLoS One* 9, e99604. doi: 10.1371/journal.pone.0099604

Pathoumthong, P., Zhang, Z., Roy, S.J., and El Habti, A. (2023). Rapid non-destructive method to phenotype stomatal traits. *Plant Methods* 19**,** 36. doi: 10.1186/s13007-023-01016-y

Peakall, R., and Smouse, P.E. (2012). GenAlEx 6.5: genetic analysis in Excel. Population genetic software for teaching and research--an update. *Bioinformatics* 28**,** 2537-2539. doi: 10.1093/bioinformatics/bts460

Peng, Y.L., Wang, Y.S., Fei, J., and Sun, C.C. (2020). Isolation and expression analysis of two novel C-repeat binding factor (CBF) genes involved in plant growth and abiotic stress response in mangrove Kandelia obovata. *Ecotoxicology* 29**,** 718-725. doi: 10.1007/s10646-020-02219-y

Pickens CN, Hester MW. (2011). Temperature Tolerance of Early Life History Stages of Black Mangrove *Avicennia germinans*: Implications for Range Expansion. *Estuaries and Coasts* 34, 824-830.doi: 10.1007/s12237-010-9358-2

Purcell, S., Neale, B., Todd-Brown, K., Thomas, L., Ferreira, M.A., Bender, D., Maller, J., Sklar, P., De Bakker, P.I., Daly, M.J., and Sham, P.C. (2007). PLINK: a tool set for whole-genome association and population-based linkage analyses. *Am J Hum Genet* 81**,** 559-575. doi: 10.1086/519795

Schneider, C.A., Rasband, W.S., and Eliceiri, K.W. (2012). NIH Image to ImageJ: 25 years of image analysis. *Nat Methods* 9**,** 671-675. doi: 10.1038/nmeth.2089

Su, W., Ye, C., Zhang, Y., Hao, S., and Li, Q.Q. (2019). Identification of putative key genes for coastal environments and cold adaptation in mangrove Kandelia obovata through transcriptome analysis. *Sci Total Environ* 681**,** 191-201.doi: 10.1016/j.scitotenv.2019.05.127

Sun, M.M., Liu, X., Huang, X.J., Yang, J.J., Qin, P.T., Zhou, H., Jiang, M.G., and Liao, H.Z. (2022). Genome-Wide Identification and Expression Analysis of the NAC Gene Family in Kandelia obovata, a Typical Mangrove Plant. *Curr Issues Mol Biol* 44**,** 5622-5637. doi: 10.3390/cimb44110381

Waadt, R., Seller, C.A., Hsu, P.K., Takahashi, Y., Munemasa, S., and Schroeder, J.I. (2022). Plant hormone regulation of abiotic stress responses. *Nat Rev Mol Cell Biol* 23**,** 680-694. doi: 10.1038/s41580-022-00479-6

Wang H, Blakeslee JJ, Jones ML, Chapin LJ, Dami IE. (2020). Exogenous abscisic acid enhances physiological, metabolic, and transcriptional cold acclimation responses in greenhouse-grown grapevines. *Plant Sci* 293, 110437. doi: 10.1016/j.plantsci.2020.110437

Wright S. (1978). Evolution and the genetic of population, variability within and among natural populations. *Chicago: University of Chicago Press* 4, 213–220.

Yang, S., Liu, X., Deng, R.-J., Chen, Q.-X., Wang, J.-W., and Lu, X. (2020). Geographic variations of hypocotyl and seedling growth traits for *Kandelia obovata* with different provenances. *Chinese Journal of Ecology* 39**,** 1769-1777. doi: 10.13292/j.1000-4890.202006.003

Zhao, C.P., Jia, M.M., Zhang, R., Wang, Z.M., Mao, D.H., Zhong, C.R., and Guo, X.X. (2024). Distribution of Mangrove Species Kandelia obovata in China Using Time-series Sentinel-2 Imagery for Sustainable Mangrove Management. *Journal of Remote Sensing* 4**,** 15. doi: 10.34133/remotesensing.0143

Zhao, Y., Zhong, Y., Ye, C., Liang, P., Pan, X., Zhang, Y.Y., Zhang, Y., and Shen, Y. (2021). Multi-omics analyses on Kandelia obovata reveal its response to transplanting and genetic differentiation among populations. *BMC Plant Biol* 21**,** 341. doi: 10.1186/s12870-021-03123-1

Zhang, J., Fan, T., Cai, X., Ouyang, S., Yang, J., Song, Z., Zhang, W., Wang, Y., Zhu, Y., and Nan, P. (2024). Comparative transcriptomic and metabolomic analysis of two related *Kandelia obovata* populations in response to cold wave. *Phenomics* accepted. doi: 10.1007/s43657-024-00204-7

Zhou, H., Hua, J., Zhang, J., and Luo, S. (2022). Negative Interactions Balance Growth and Defense in Plants Confronted with Herbivores or Pathogens. *J Agric Food Chem* 70**,** 12723-12732. doi: 10.1021/acs.jafc.2c04218

Zhu Hong, Lin Hai-Jiao, Yang Le, Li He-Peng, Yue Chun-Lei, Jiang Bo. (2021). Geographical distribution pattern and environmental explanation of *Kandelia obovata* Sheue, H.Y. Liu & J. Yong populations along the Southeast coast of China. *Plant Science Journal* 39, 476-487. doi: 10.11913/PSJ.2095-0837.2021.50476
